# Gene Expression Profiling in Fibromyalgia Indicates an Autoimmune Origin of the Disease and Opens New Avenues for Targeted Therapy

**DOI:** 10.3390/jcm9061814

**Published:** 2020-06-10

**Authors:** Marzia Dolcino, Elisa Tinazzi, Antonio Puccetti, Claudio Lunardi

**Affiliations:** 1 Department of Medicine, University of Verona, Piazzale L.A. Scuro 10, 37134 Verona, Italy; marziadolcino@gmail.com (M.D.); elisa.tinazzi@univr.it (E.T.); 2 Department of Experimental Medicine, Section of Histology, University of Genova, Via G.B. Marsano 10, 16132 Genova, Italy; antonio.puccetti@unige.it

**Keywords:** fibromyalgia, long non-coding RNA, signaling pathway, protein-protein (PPI) network, gene module

## Abstract

Fibromyalgia is a chronic disorder characterized by widespread pain and by several non-pain symptoms. Autoimmunity, small fiber neuropathy and neuroinflammation have been suggested to be involved in the pathogenesis of the disease. We have investigated the gene expression profile in peripheral blood mononuclear cells obtained from ten patients and ten healthy subjects. Of the 545,500 transcripts analyzed, 1673 resulted modulated in fibromyalgic patients. The majority of these genes are involved in biological processes and pathways linked to the clinical manifestations of the disease. Moreover, genes involved in immunological pathways connected to interleukin-17 and to Type I interferon signatures were also modulated, suggesting that autoimmunity plays a role in the disease. We then aimed at identifying differentially expressed Long non-coding RNAs (LncRNAs) functionally connected to modulated genes both directly and via microRNA targeting. Only two LncRNAs of the 298 found modulated in patients, were able to target the most highly connected genes in the fibromyalgia interactome, suggesting their involvement in crucial gene regulation. Our gene expression data were confirmed by real time PCR, by autoantibody testing, detection of soluble mediators and Th-17 polarization in a validation cohort of 50 patients. Our results indicate that genetic and epigenetic mechanisms as well as autoimmunity play a pivotal role in the pathogenesis of fibromyalgia.

## 1. Introduction

Fibromyalgia (FM) is as a chronic widespread pain condition associated with several symptoms such as fatigue, sleep and cognitive disturbances, dysautonomia, depression and a variety of somatic symptoms [1,2].

FM is a multifaceted condition, as reported in the American College of Rheumatology (ACR)-2010 diagnostic criteria, which changed the definition from a “peripheral pain-defined disease” to a “systemic symptom-based disease” [3]. The diagnosis and management of FM as well as its definition have been updated by the Canadian guidelines in 2012 [4] and more recently by Arnold et al. [5] and by Salaffi et al. [6]. In particular symptoms reported in FM include both systemic symptoms, such as fatigue, fever, diffuse stiffness, myalgias and muscle weakness, loss of appetite, and symptoms related to organ involvement, such as involvement of both peripheral and central nervous system, glandular involvement with dry eyes and dry mouth, gastrointestinal symptoms or urinary manifestations.

The lack of specific biomarkers for the diagnosis of FM is a well-known diagnostic problem [3] and the elimination of the tender point examination from the modern criteria makes the diagnosis of FM even more difficult.

Since the pathogenetic basis of the disease is still unclear, the treatment has mainly targeted the widespread pain and depression.

Different hypotheses have been proposed to explain the pathogenesis of FM; recently two mechanisms have been suggested: neuroinflammation and autoimmunity [7]. Indeed, neuroinflammation has been suggested to play a fundamental role in the pathogenesis of the disease. In this setting it has recently been demonstrated the presence of high levels of cytokines and chemokines in the cerebrospinal fluid as well as the presence of a strong activation of microglia and lymphocytes both at central and peripheral nervous system levels [8,9]. 

The hypothesis that autoimmunity may be involved in the pathogenesis of FM is based on different features, such as the strong association with autoimmune diseases [10,11], the relation between the onset of fibromyalgic symptoms and trauma or infections, that are connected also to the development of autoimmunity, a strong female predominance and the detection of autoantibodies such as anti-smooth muscle and anti-striated muscle autoantibodies. In addition FM may accompany subclinical stages of some autoimmune diseases such as Hashimoto thyroiditis and Sjogren’s syndrome and in these cases antithyroperoxidase and anti-nuclear antibodies have been detected in 30% of the patients [7,12,13,14]. 

Moreover, a strong association of human leukocyte antigen (HLA) genes connected to autoimmunity and activation of the adaptive immune system with the involvement of both B and T lymphocytes have been recently reported in FM [15,16]. Recently, a bioinformatic analysis has highlighted a subset of differently up or down-regulated genes and microRNAs in FM defining a group of genes potentially involved in FM and significantly associated to musculoskeletal, mental and immune system disorders [17].

Although the above mentioned aspects are highly suggestive for an autoimmune component in the pathogenesis of FM [7], a link between autoimmunity and the disease has not yet been definitively proven. 

Therefore our aim was to better clarify the presence of an autoimmune component in fibromyalgic patients. In this regard, we used a whole gene expression analysis to identify transcriptional profiles associated to the vast array of FM features. Moreover we have evaluated epigenetic aspects that may be involved in the pathogenesis of the disease. Indeed we have applied this procedure in the study of other immune-mediated diseases and have identified non coding RNAs, in particular, long non coding RNAs (LncRNAs) as one of the epigenetic mechanisms able to modulate gene expression both directly and indirectly through microRNAs [18,19,20]. 

Indeed, using this approach, we identified immunological pathways that indicate the presence of an autoimmune component in the pathogenesis of FM. Moreover, these findings are further sustained by autoantibody testing, by detection of soluble mediators and by Th-17 polarization in a training group of 10 fibromyalgic patients and in a validation cohort of 50 patients. 

## 2. Materials and Methods

### 2.1. Patients

We enrolled 10 patients affected by FM (9 females and 1 male, mean age 45 ± 16.5 years), diagnosed according to the American College of Rheumatology 2010 criteria [3], and 10 age and sex matched healthy controls (9 females and 1 male, mean age 42 ± 18.5) for the gene expression analysis.

Fifty fibromyalgic patients (48 females and 2 males, mean age 49 ± 20.5 years) and 50 healthy age and sex matched controls (47 females and 3 males, mean age 47 ± 21.5 years) were used as validation groups.

In the group of fibromyalgic patients we included only subjects that fulfilled the ACR diagnostic criteria; we considered as exclusion criteria the positive history of malignancies, the presence of active infection as well as of autoimmune or autoinflammatory disease. The control group included subjects without any history of chronic as well as autoimmune or neoplastic disease.

Peripheral blood was obtained from patients who were not under any treatment, in particular steroids, for at least 2 weeks. 

A written informed consent was obtained from all the participants of the study and the study protocol was approved by the Ethical Committee of the Azienda Ospedaliera Universitaria Integrata di Verona. All the investigations have been performed according to the principles contained in the Helsinki declaration.

### 2.2. PBMCs Isolation

Peripheral blood mononuclear cells (PBMCs) were obtained from 60 healthy donors (training plus validation group) and 60 patients affected by FM (training plus validation group) through a density-gradient centrifugation on Lymphoprep (Nycomed Pharma, Oslo, Norway) at 800 ×*g*. Cells were washed twice with PBS and counted using acridine orange (Thermo Fisher Scientific, Waltham, MA, USA), considering only viable cells for fluorescence-activated cell sorting (FACS) analyses.

### 2.3. Microarray Analysis

Blood sample collection was performed using BD Vacutainer K2EDTA tubes (Becton Dickinson, Franklin Lakes, NJ, USA) and 21-gauge needles.

PBMCs isolation was carried out by Ficoll-HyPaque (Pharmacia Biotech, Baie d’Urfé QC, Canada) gradient centrifugation. Patients and controls had a similar PBMCs distribution. Total RNA was extracted from PBMCs (10^7^ cells) using an miRNeasy mini kit (Qiagen GmbH, Hilden, Germany). cRNA preparation, sample hybridization and scanning were performed following the protocols provided by Affymetrix (Santa Clara, CA, USA), using a Cogentech Affymetrix microarray unit (Campus IFOM IEO, Milan, Italy). All samples were hybridized on a Human Clariom D (Thermo Fisher Scientific, Waltham, MA, USA) gene chip. Signal intensities were analyzed with Transcriptome Analysis Console (TAC) 4.0 software (Applied Biosystems, Foster City, CA, USA).

Using the Human Clariom D arrays, more than 540,000 human transcripts can be interrogated, starting from as little as 100 pg of total RNA. The signal intensity was background-adjusted, normalized and log-transformed using the signal space transformation (SST)—robust multi-array average algorithm (RMA).

Differentially expressed genes that showed an expression level at least 1.5-fold different in the test sample versus a control sample at a significant level (false discovery rate (FDR) corrected *p*-value ≤ 0.01) were chosen for final consideration. 

Long non-coding RNAs have been identified based on the annotations provided by Thermofisher (thermofisher.com) and included in the GeneChip Array Annotation Files. These information have been confirmed in reference databases using public identifications codes provided by Thermofisher.

Target annotations of long non-coding RNAs were retrieved using NPInter v4.0 (http://bigdata.ibp.ac.cn/npinter4) [21], selecting only lncRNAs interactions, that have been already experimentally validated by high-throughput experimental technologies such as, photoactivatable ribonucleoside-enhanced crosslinking and immunoprecipitation, RNA interference and RT-PCR. The list of gene targets of microRNAs (miRNAs) that are targeted by lncRNAs was gathered from the FunRich database (http://www.funrich.org/) [22].

### 2.4. Protein-Protein Interaction (PPI) Network Construction and Network Clustering

The PPI network was constructed upon the experimentally validated protein‒protein interactions using STRING (Search Tool for the Retrieval of Interacting Genes) version 10.5 (http://string-db.org/) [23]. Network topological analysis was performed using Cytoscape software (http://www.cytoscape.org/) [24]. High-flow areas (highly connected regions) of the network (modules) were detected using the MCODE plugin of Cytoscape (*k*-core = 4 and node score cutoff = 0.2).

### 2.5. Gene Functional Classification and Enrichment Analysis

Genes were functionally classified into biological processes (BPs) according to the gene ontology (GO) annotations (http://www.geneontology.org/) [25] by the Panther expression analysis tools (http://pantherdb.org/) [26].

Pathway classification and enrichment (Bonferroni corrected *p-*value ≤ 0.05) analysis were achieved with FunRich.

### 2.6. Real-Time RT-PCR

Total RNA was isolated from PBMC using TRIzol Reagent (Invitrogen, Carlsbad, CA, USA), following the manufacturer’s instructions. PCR was performed by following the methods of Dolcino et al. [27].

In Supplementary Table S1 the primers used for the validation of the selected LncRNAs are listed.

### 2.7. FACS Analysis

Cell samples were treated according to the methods by Dolcino et al. [28]. Cells were stimulated over-night with Dynabeads Human T-Activator CD3/CD28 (Life Technologies, Carlsbad, CA, USA). The detection of IL-17 production was analyzed using the IL-17 Secretion Assay (Miltenyi Biotec, Bergisch Gladbach, German), following the manufacturer’s instruction as described in the methods of Dolcino et al. [28].

### 2.8. Detection of Soluble Mediators in Sera of FM Patients and Healthy Controls

Serum levels of TGF-beta, IL-6, IL-23, IL-21, IL-17, TNF, IL-10, IL-8, IL-1, IL-2 and IL-4 were detected using commercially available ELISA kits according to the manufacturer’s instructions, in the training group (10 FM patients and 10 healthy subjects) and in the validation group (50 FM patients and 50 healthy subjects). ELISA kits for the detection of TGF-beta, IL-6, IL-23, IL-17, TNF, IL-10, IL-8, IL-1, IL-2 and IL-4 were purchased by R&D Systems whereas ELISA kits for the detection of IL-21 were purchased by Invitrogen. 

Serum levels of neuroinflammation-associated soluble molecules were detected using V-PLEX Neuroinflammation Panel 1 Human Kit (Meso Scale Diagnostics, Rockville, MD, USA).

### 2.9. Sjögren’s Autoantibodies Detection in FM patients’ Sera

The presence of the classic Sogrens’ autoantibodies (anti-SSA/Ro, anti-SSB/La, Anti-Nuclear Antibodies, Rheumatoid Factor) and the early tissue specific autoantibodies (TSAs) (SP-1, CA6, PSP) was evaluated by Enzyme-Linked Immuno Assay (ELISA) using reagents purchased from Quest Diagnostics (Tampa, FL, USA) and Labcorp (Clearwater, FL, USA) both in the training group and in the validation group of patients.

Moreover, we assessed by ELISA test the antiserotonin, antiganglioside and antiphospholipid antibodies concentration in FM patients’ sera using reagents purchased by MyBiosource.

### 2.10. Statistical Analysis

Statistical testing was performed using SPSS Statistics 2 software (IBM, Armonk, NY, USA). Data obtained from the analysis of the soluble mediators and from the analysis of IL-17-positive CD4+ T cells in PBMCs were analyzed using the Student’s unpaired t-test.

Data obtained from the analysis of soluble mediators and RT-PCR validation were analyzed using the non-parametric Mann–Whitney test.

## 3. Results

### 3.1. High-Throughput Gene Expression Profiling in Peripheral Blood Mononuclear Cells of FM Patients

With the aim of identifying genes potentially involved in FM pathogenesis, we contemporaneously profiled the expression of more than 540,000 human transcripts, including those ascribed to more than 50,000 long non-coding RNAs (lncRNAs), in a training group of twenty PBMCs samples (10 FM and 10 healthy subjects). 

Transcriptional profiles of FM patients and healthy subjects were compared and, after a robust filtering procedure (FDR-corrected *p*-value ≤ 0.01 and fold change ≥ |1.5|), 1673 significantly differentially expressed coding genes were selected. 

**Figure 1:**
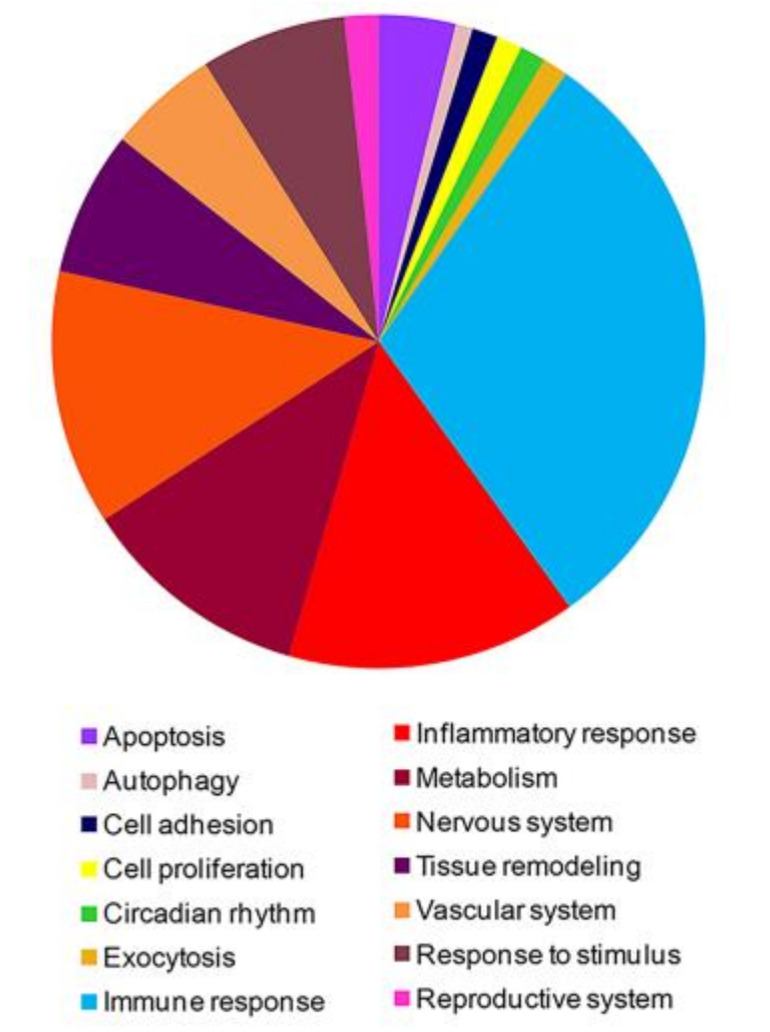
Graphical representation of gene ontology biological processes in which genes modulated in fibromyalgia (FM) patients are involved.

The 1673 differentially expressed genes (DEGs) were thoroughly examined and functionally classified by gene ontology (http://www.geneontology.org/) and, meaningful biological processes (BP) that were significantly enriched (*p* < 0.05) in modulated genes were highlighted. A large number of enriched BP strictly associated to the array of FM-associated clinical manifestations were selected and graphically represented in Figure 1. A detailed description of the enriched functional classes is reported in Supplementary Table S2. Meaningful enriched BPs were related to apoptosis, autophagy, circadian rhythm, exocytosis, immune response, inflammatory response, metabolism, nervous system, tissue remodeling, vascular system, response to stimulus and reproductive system. Interestingly, a large amount of genes significantly enriched BPs of the immune response and several of them were ascribed to Th-17 and Type I interferon signature (Table 1). All the 1673 were then submitted to a pathway enrichment analysis (Bonferroni corrected *p*-value ≤ 0.05) to highlight important signaling networks in which modulated genes were involved. These pathways included, for example, signaling that regulate vascular biology (i.e., VEGF and VEGFR signaling; endothelins signaling), immune response (IL2 signaling, TCR signaling, IL23 signaling and CD40/CD40L signaling), inflammatory response, endocrine system, circadian rhythm, apoptosis, inflammatory response, metabolism, pain perception and transmission and neuronal system (Supplementary Table S3).

**Figure 2:**
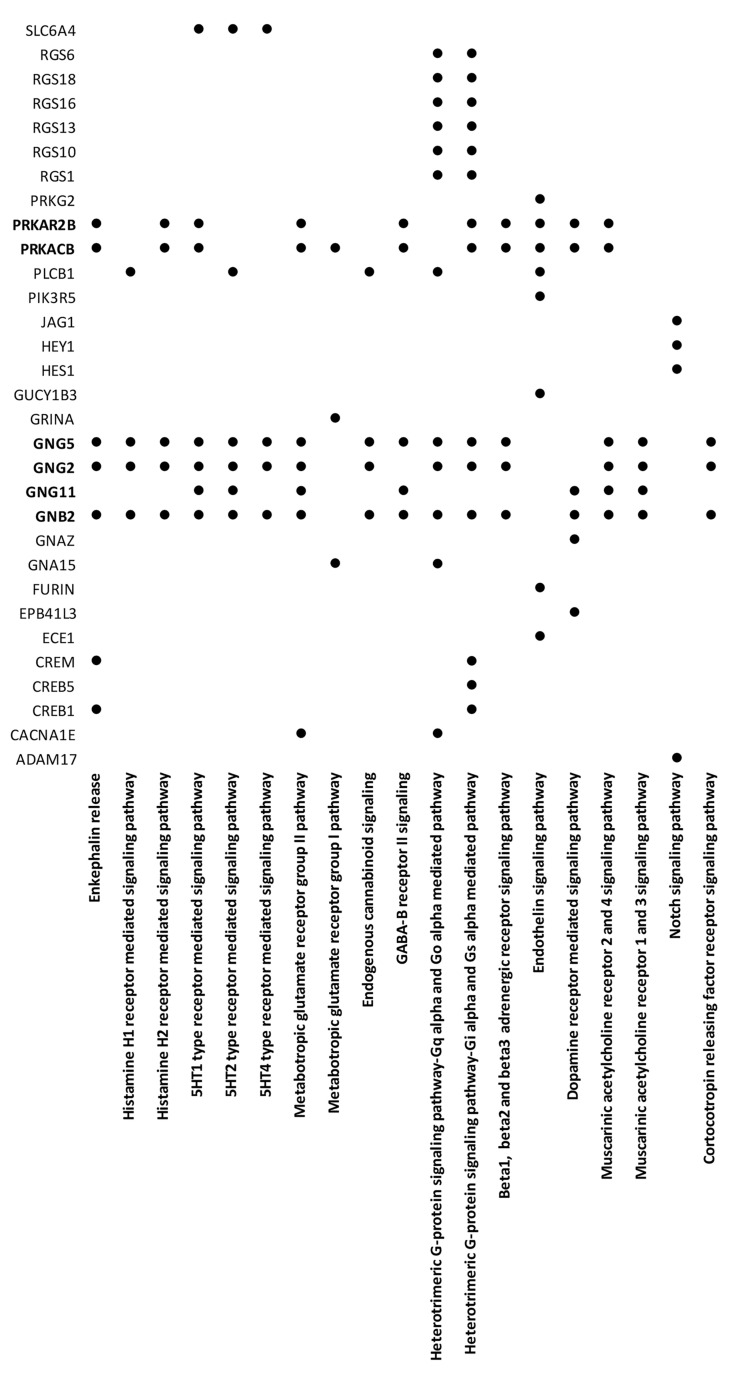
Genes modulated in FM patients that are involved in signaling pathways that regulate pain perception and transmission. Pathways associated to pain perception and transmission are listed vertically.

Noteworthy, we identified a group of genes that were simultaneously involved in different signaling pathways that regulate pain perception and transmission (Figure 2).

### 3.2. PPI Network and Modular Analysis of Genes Modulated in FM

It is a matter of fact that the modulation of highly connected genes can have a more prominent effect in the disease pathogenesis than the fluctuation of genes that are not functionally connected, we therefore, performed a network analysis to identify all the differentially expressed genes that were functionally connected and to prioritize transcripts that may have a role in FM pathogenesis.

A protein–protein interaction (PPI) network, that included all the experimentally validated functional interactions among the protein products of the 1673 modulated genes (nodes) in FM was constructed and, 1492 DEGs resulted to be functionally connected (Supplementary Figure S1).

We then performed a modular analysis to identify areas in which the most highly interacting genes were connected and we could highlight six gene modules that were most likely involved in the disease pathogenesis (Supplementary Table S4).

**Figure 3:**
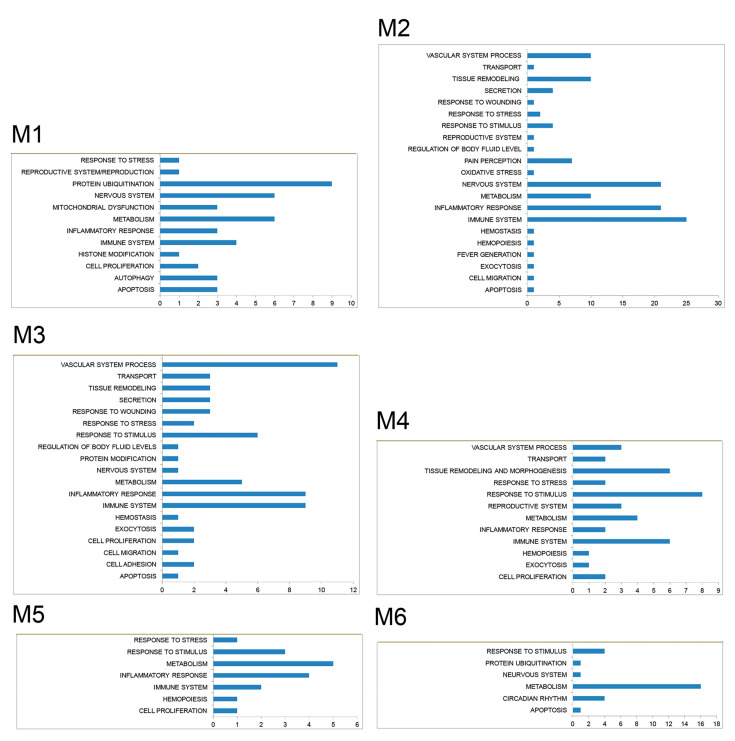
Significantly (*p* < 0.01) enriched biological processes in which are distributed genes modulated in FM patients that are included in the six modules of highly connected genes.

**Figure 4:**
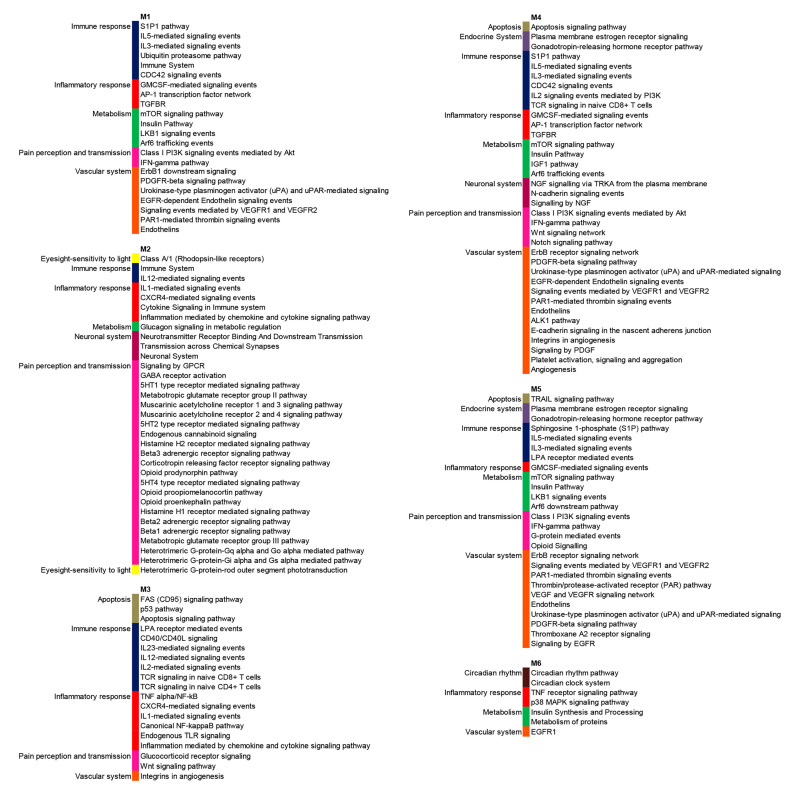
Significantly (*p* < 0.01) enriched signaling pathways in which are distributed genes modulated in FM patients that are included in the six modules of highly connected genes.

The enrichment analysis of module-associated genes showed that these genes were involved in almost all the relevant processes and pathways in which the vast majority of modulated genes in FM (Figure 3 and Figure 4) is involved.

### 3.3. High-Throughput Long Non-Coding RNA Expression Profiling in Peripheral Blood Mononuclear Cells of Patients with FM

The above described filtering approach (FDR-corrected *p*-value ≤ 0.01 and fold change ≥ |1.5|) was applied to non-coding transcripts and we found a significant modulation of 298 long non-coding RNAs (LncRNAs) in FM samples when compared to samples obtained from the control group. The 298 modulated LncRNAs were filtered retaining only those transcripts for which experimentally validated microRNA (miRNA) targets have been already annotated in NPinter and, among these, only lncRNAs with fold change (FC) above or equal to four (Supplementary Table S5). By this criterion, five lncRNAs were filtered including: CTD-2651B20.6, RP1-151F17.1, AC009299.3, RP11-283I3.6 and RP11-747H7.3 (Table 2).

We, therefore, examined all genes controlled by the miRNA targets of the six lncRNA that were experimentally validated by high-throughput technologies (see the Materials and Methods section) and we picked out only those microRNAs that could directly control FM-DEGs (Supplementary Table S6). We thus realized that all the selected lncRNAs, but RP11-283I3.6, could regulate genes differentially expressed in FM patients either directly or via miRNA interactions that have been experimentally validated (Table 2) and for this reason RP11-283I3.6 LncRNA was excluded from further analysis.

To prioritize LncRNAs with the highest probability to exert a more pronounced effect on FM transcriptome, we verified whether the five selected LncRNAs could control highly interacting genes in the FM network and we observed that only AC009299.3 and RP11-747H7.3 could targets genes in all the six modules (Table 2 and Supplementary Table S7). Based on this evidence our analysis was narrowed down on these two LncRNAs.

We verified that AC009299.3 (ENSG00000235724; genecards.org/cgi-bin/carddisp.pl?gene=ENSG00000235724, accessed on 3 January 2020) encoded for the antisense sequence of the TANK gene while, RP11-747H7.3 (ENSG00000260711; grch37.ensembl.org/Homo_sapiens/Gene/Summary?g=ENSG00000260711;r=14:92223103-92226142;t=ENST00000565058, accessed on 3 January 2020) encoded for an intronic sequence of CATSPERB gene, and that both these transcripts had not yet been functionally characterized.

The examination of targeted highly connected genes included in the six modules showed that the two newly identified LncRNAs regulated crucial transcripts related to immune response and to clinical manifestations of FM (or to particular features of FM). Indeed, several targeted genes were involved in innate immune response (i.e., CLP1 and TRAF3) and in adaptive immune response (i.e., CTPS1, operating in T and B cell proliferation, BCL2L11 related to B and T cell homeostasis and BET1 expressed by T regulatory cells). Interestingly, CCL7, CEBPB, IL10, S1PR1 and SOCS1 belonged to genes of the Th-17 signature and IL10, SOCS1 and TRAF3 were members of the Type 1 interferon signature. Moreover, SOCS1, TNFRSF1B, THBS1, HSPA5 and IL10 were involved in inflammatory response.

A group of targeted highly connected genes were involved in nervous system biology including ZEB1 (central nervous system development), GRIA1 (chemical synaptic transmission), CRY2 (circadian clock system), MEIS1 (regulation of neuron differentiation), KIF2A (nervous system development), DDIT3 (positive regulation of neuron apoptotic process), SDC2 (dendrite morphogenesis), GNA13 (G-protein signaling in brain development) and GNG5 that is an important member of pain transmission signaling pathways (Figure 2).

To a deeper dissection of targeted genes included in the six modules, we interrogated GeneCards (www.genecards.org) to inspect all possible diseases that have been previously associated to these transcripts and we found that several genes were involved in cardiovascular, osteoarticular, autoimmune, neuronal and muscular diseases. Of note, some of the targeted genes have been previously associated to intellectual disability, depression, myopathy, Sjogren’s syndrome and chronic fatigue syndrome (Table 3).

We next compared all the signaling pathways that were significantly enriched in FM transcriptome to the signaling networks that we found enriched in the Rheumatoid arthritis dataset that we previously examined [24] and we found that a large numbers of gene signatures were commonly enriched in the two datasets (Supplementary Table S8).

The level of expression of the five selected LncRNA (Supplementary Figure S2) was validated by RT-PCR and, statistically significant differences (*p* < 0.0001) between patients and healthy subjects were found in the expression levels of all the tested transcripts thus confirming the gene array results.

### 3.4. Frequency of IL-17 Positive CD4+ T Cells in PBMCs from Patients with FM

The intracellular expression of the IL-17 cytokine was assessed by flow cytometry, in PBMCs from both training and validation group (60 FM patients and 60 healthy subjects). We found a higher amount of IL-17-producing CD4+ T cells among the PBMCs of patients with FM compared with healthy controls.

The mean values obtained in 60 FM PBMC were 1.3% ± 0.15 versus 0.3% ± 0.11 (*p* < 0.0001).

### 3.5. Detection of Soluble Mediators in FM Sera

The gene expression analysis was complemented by the detection of some soluble mediators in the sera of patients with FM.

We have chosen to test the levels of Th-17 related cytokines and we found a higher amount of cytokines that promote the Th17 lineage differentiation (TGF-beta and IL-6) and its survival and expansion (IL-21 and IL-23) and of IL-17 in FM patients compared to healthy subjects when both the training and the validation group were tested *(p* < 0.0001) (Supplementary Figure S3).

In FM patients the serum levels of TNF, IL-10, and IL-8 were not significantly different (p-value of 0.1210, 0.3738, and 0.1825, respectively) from those detected in the serum of healthy subjects whereas, IL-1, IL-2 and IL-4 were expressed at a slightly higher level in FM patients’ sera than in the sera of healthy controls (*p-*value < 0.01, <0.0001 and <0.0001 respectively) (Supplementary Figure S3).

We, moreover, assessed the serum levels of soluble factors associated to neuroinflammation and we found a significantly increased concentration of Tie-2, SAA and TARC in FM patients compared to healthy subjects (*p* < 0.0001) (Supplementary Figure S3).

### 3.6. Autoantibodies Detection in FM Patients’ Sera

Of the 60 patients (training and validation group) who were evaluated for both the early and classic SS markers, 18 (30%) tested positive for SS autoantibodies and 15 (25%) tested positive for the early tissue specific autoantibodies only.

Moreover, we assessed the antiserotonin, antiganglioside and antiphospholipid antibodies concentration in FM patients’ sera and, we found increased levels (*p* < 0.0001) of these antibodies in 21%, 18% and 15% of patients after comparison to healthy subjects.

## 4. Discussion and Conclusions

In this work, for the first time we provide a comprehensive analysis of the transcriptome and interactome in patients affected by FM.

We have successfully applied this approach to study complex diseases such as systemic sclerosis, psoriatic arthritis and Behcet disease [18,19,20]. The results we report here dissect different aspects of FM, shedding a new light on the pathogenesis of this multifaceted disorder.

In particular we have better clarified the role of the immune system in FM and have provided evidence for an autoimmune component in the pathogenesis of the disease, since FM gene expression profiles are characterized by a dual gene signatures (Th-17 and Type I interferon); combined presence of these two signatures is typical of autoimmune diseases, as demonstrated by other investigators including ourselves [28,29,30,31,32]. Of note are the higher levels of circulant IL-17 producing CD4+ T cells and of serum cytokines such as TGF-beta, IL-6, IL-21 and IL-23 that promote Th17 differentiation, survival and expansion in fibromyalgic patients, thus confirming the gene signature and the presence of an ongoing inflammatory process.

We have also identified two yet uncharacterized LncRNAs that both directly and through their miRNA targets, regulate networks of genes that are involved in multiple aspects of FM and, above all, are able to regulate important immunological aspects that are connected to the pathogenesis of the disease.

Since it is well known that the regulation of highly connected genes can have a more pronounced impact on the development of a disease than the targeting of transcripts that show no functional interactions, we wanted to verify that the two selected LncRNAs could regulate highly interacting genes in the FM transcriptome.

With this aim, the canonical gene expression analysis was implemented by a network analysis and we dissected the whole pattern of the experimentally validated functional interactions among the modulated transcripts, tracing with a good confidence all LncRNAs-miRNAs-genes interplays.

By this approach we could highlight that the two selected LncRNAs have the ability to target the majority of the highly connected genes in the FM interactome, which are believed to be crucially involved in the onset and main features of the disease. Indeed, it is important to point out that a group of targeted highly connected genes are involved in nervous system biology and have been previously associated to depression, Sjogren’s syndrome, osteoarticular, autoimmune, neuronal and muscular diseases.

Our results were validated in an extended cohort of patients and confirmed by different tests including detection of circulant autoantibodies in a percentage of patients and of soluble mediators and of IL-17 production as reported above. Of note serum levels of TNF-alpha, IL-10 and IL-8 were not statistically different in patients compared to healthy subjects. This aspect may be a particular feature of the disease and deserves to be further confirmed.

We believe that this work represents an important contribution to the comprehension of the pathogenesis of FM and that it can have remarkable clinical implications.

Indeed, the identification of the two epigenetic regulators that control crucial molecular pathways involved in FM, and the identification of an autoimmune component in the origin of the disease can lead to more effective therapeutic choices especially for the treatment of fibromyalgic patients who have no benefit from conventional pain control therapies.

Further studies are needed in an extended cohort of patients, to confirm our data on the importance of epigenetic factors and of autoimmune aspects in the pathogenesis of FM.

## Figures and Tables

**Table 1:** Th-17 and Type I interferon related genes that are modulated in FM patients.

ID	*p-*Value	Gene Symbol	Description
**Th-17 Related Genes**			
TC0100010686.hg.1	0.005	CACYBP	calcyclin binding protein
TC0200010980.hg.1	<0.001	CCL20	chemokine (C-C motif) ligand 20
TC1700007558.hg.1	0.005	CCL7	chemokine (C-C motif) ligand 7
TC0 × 00008540.hg.1	0.004	CD40LG	CD40 ligand
TC2000007682.hg.1	0.001	CEBPB	CCAAT/enhancer binding protein (C/EBP). beta
TC1200011177.hg.1	0.008	IFNG	interferon. Gamma
TC0100017107.hg.1	0.001	IL10	interleukin 10
TC0200013916.hg.1	0.001	IL1B	interleukin 1 beta
TC0700006890.hg.1	<0.001	IL6	interleukin 6
TC2000007514.hg.1	0.002	MMP9	matrix metallopeptidase 9
TC0400008271.hg.1	0.002	NFKB1	nuclear factor of kappa light polypeptide gene enhancer in B-cells 1
TC0100009241.hg.1	0.005	S1PR1	sphingosine-1-phosphate receptor 1
TC1600009395.hg.1	0.006	SOCS1	suppressor of cytokine signaling 1
TC0600007596.hg.1	<0.001	TNF	tumor necrosis factor
**Type I Interferon Related Genes**			
TC0600007495.hg.1	0.003	HLA-A	major histocompatibility complex. class I. A
TC0200014772.hg.1	0.008	IFIH1	interferon induced. with helicase C domain 1
TC0100017107.hg.1	0.001	IL10	interleukin 10
TC0700006890.hg.1	<0.001	IL6	interleukin 6
TC0400012606.hg.1	0.002	IRF2	interferon regulatory factor 2
TC1400010584.hg.1	0.005	IRF9	interferon regulatory factor 9
TC0700008875.hg.1	<0.001	MET	MET proto-oncogene. receptor tyrosine kinase
TC1500007833.hg.1	<0.001	PML	promyelocytic leukemia
TC0900012231.hg.1	0.0004	SHB	Src homology 2 domain containing adaptor protein B
TC1600009395.hg.1	0.006	SOCS1	suppressor of cytokine signaling 1
TC0 × 00007149.hg.1	0.006	TIMP1	TIMP metallopeptidase inhibitor 1
TC0 × 00006625.hg.1	<0.001	TLR7	toll-like receptor 7
TC1400008362.hg.1	0.008	TRAF3	TNF receptor-associated factor 3
TC1900009627.hg.1	0.005	TYK2	tyrosine kinase 2
TC0600008109.hg.1	0.003	VEGFA	vascular endothelial growth factor A

**Table 2:** Selected long non coding RNAs (LncRNAs) modulated in FM patients.

LncRNAs	FC	Targeted miRNAs	Targeted FM-DEGs	Targeted Module-Associated FM-DEGs	Targeted Modules
**CTD-2651B20.6**	5.23	2	12	4	M3 M5 M6
**RP1-151F17.1**	4.65	15	75	16	M2 M3 M4 M5 M6
**AC009299.3**	−4.52	29	183	38	M1 M2 M3 M4 M5 M6
**RP11-283I3.6**	−5.05	1	none	none	none
**RP11-747H7.3**	−9	8	57	12	M1 M2 M3 M4 M5 M6

**Table 3:** Module associated genes and main disorders to which they can be associated.

Module Associated Gene	Module	Diseases (GeneCards)
SOCS1	1	coronary heart disease 1, myeloproliferative neoplasm, b-cell lymphoma, placental insufficiency, multiple sclerosis
ASB6	1	None
LMO7	1	cardiomyopathy, dilated, 1dd
UBE2H	1	pontocerebellar hypoplasia, Type 7
TNFSF11	2	bone disease, depersonalization disorder, psoriatic arthritis
TNFRSF1B	2	juvenile rheumatoid arthritis, psoriatic arthritis, Guillain-Barre syndrome
CCL3	2	aseptic meningitis, bone disease, rheumatoid arthritis, arthritis, demyelinating disease, immune deficiency disease
GNG5	2	echolalia
S1PR1	2	autoimmune disease of central nervous system, multiple sclerosis
IL10	3	rheumatoid arthritis, arthritis, ulcerative colitis, rheumatic heart disease, chronic fatigue syndrome
THBS1	3	thrombotic thrombocytopenic purpura, thrombocytopenia
TRAF3	3	encephalitis, lymphoma, Hodgkin, classic, myeloma, multiple
KIF2A	3	cortical dysplasia, complex, with other brain malformations 3, Walker-Warburg syndrome
CCL7	3	nephrogenic systemic fibrosis, demyelinating disease, inflammatory bowel disease, multiple sclerosis
RCC2	3	spermatogenic failure 5, renal cell carcinoma, nonpapillary
BORA	3	branchiootic syndrome
CEP97	3	spinocerebellar ataxia 11, nephronophthisis, bardet-biedl syndrome
NHLRC2	3	fibrosis, neurodegeneration, and cerebral angiomatosis
HSPA5	4	rheumatoid arthritis, wolfram syndrome 1
CDKN1A	4	Tumors
BCL2L11	4	autoimmune lymphoproliferative syndrome, leukemia
PPP2R2A	4	syndromic intellectual disability
GRIA1	4	depression
ZEB1	4	congenital hypothyroidism, corneal endothelial dystrophy
BET1	4	leukodystrophy, hypomyelinating
CWC22	4	bruxism
CLP1	4	pontocerebellar hypoplasia
SEC23IP	4	Warrensburg’s syndrome
CSTF1	4	None
PPIL1	4	None
TFRC	5	immunodeficiency 46, iron deficiency anemia
EXOSC10	5	systemic scleroderma, collagen disease
FAS	5	lymphoproliferative syndrome, Sjogren syndrome
CCNH	5	capillary malformation-arteriovenous malformation 1, weber syndrome, xeroderma pigmentosum
WASL	5	Wiskott–Aldrich syndrome, x-linked recessive disease
CTSB	5	acute pancreatitis
GNA13	5	Burkitt lymphoma, retinitis pigmentosa 14
SURF4	5	spinocerebellar ataxia, autosomal recessive, with axonal neuropathy 2, macular degeneration, age-related
HBEGF	5	perinatal necrotizing enterocolitis, interstitial cystitis, bladder disease, urinary tract obstruction
MEIS1	5	myeloid leukemia, restless legs syndrome
SDC2	5	autism, rheumatic disease, fibrodysplasia ossificans progressiva
RPS3	6	Warrensburg syndrome
CEBPB	6	alk-positive anaplastic large cell lymphoma, myxoid liposarcoma, retinoblastoma, acute promyelocytic leukemia,
DDIT3	6	liposarcoma, fatty liver disease, lipomatosis, multiple
CTPS1	6	nemaline myopathy, immunodeficiency, sleeping sickness
CRY2	6	major depressive disorder, advanced sleep phase syndrome, nephronophthisis, delayed sleep phase disorder

## Supplementary Materials

The following are available online at https://www.mdpi.com/2077-0383/9/6/1814/s1, Figure S1: protein-protein interaction network of modulated genes in FM patients, Figure S2: validation of selected differentially expressed LncRNAs by real time-PCR. Real-time PCR for the indicated LncRNAs were performed in healthy controls and in FM patients enrolled for the microarray analysis and in an expanded cohort of healthy subjects (50 subjects), and FM patients (50 patients). Values are calculated with ΔCt method. Histograms indicate mean values; bars indicate standard deviation (SD), Figure S3: serum levels of selected molecules in the training group and in the validation group of FM patients and healthy controls. Bars indicate SD. Table S1: sequences of primers used for the detection of LNCRNAs, Table S2: biological processes that are enriched in the 1673 genes that are differentially expressed in FM patients, Table S3: signalling pathways that are enriched in the 1673 genes that are differentially expressed in FM patients, Table S4: genes modulated in FM patients that are included in the six modules, Table S5: selection of Lnc-RNAs modulated in FM patients, Table S6: miRNAs targets of the selected Lnc-RNAs, Table S7: targeted genes modulated in FM patients that are included in the six modules, Table S8: signalling pathways significantly enriched in Rheumatoid arthritis and in FM datasets.
