# Supplementary material for: Gene Expression Profiling in Fibromyalgia Indicates an Autoimmune Origin of the Disease and Opens New Avenues for Targeted Therapy
Source: J Clin Med. 2020 Jun 10;9(6):1814. doi: 10.3390/jcm9061814 (PMC7356177; doi:10.3390/jcm9061814)
Supplement: Supplementary file 1 [file jcm-09-01814-s001.zip › Supplementary table 1.pdf]

Supplementary table 1. Sequences of primers used for the detection of LncRNAs

LncRNA	Left primer	Right primer
**CTD-2651B20.6**	GAAGAGTTCAGGCGACAAGC	CCACAATGTTGTGAACTCAGG
**RP1-151F17.1**	GCAACTCCGGAAAGTTTCAA	CGGTTGCTTTCTCTGTCTCC
**AC009299.3**	GGTCTAAGCCCCAAGAAAGG	GAGATCTTCCACCACGGAAA
**RP11-283I3.6**	TATGGTGGCATACGCCTGTA	GATACTTGGGCTCCCTGTGA
**RP11-747H7.3**	CCATGTTCTTTGGGAATGCT	GGACTTCCCAGTTGCCATAA
