# Supplementary material for: Gene Expression Profiling in Fibromyalgia Indicates an Autoimmune Origin of the Disease and Opens New Avenues for Targeted Therapy
Source: J Clin Med. 2020 Jun 10;9(6):1814. doi: 10.3390/jcm9061814 (PMC7356177; doi:10.3390/jcm9061814)
Supplement: Supplementary file 1 [file jcm-09-01814-s001.zip › Supplementary table 2.pdf]

	GO biological process complete	*p-value*
**APOPTOSIS**	apoptotic process (GO:0006915)	<0.01
APOPTOSIS	programmed cell death (GO:0012501)	<0.01
APOPTOSIS	regulation of programmed cell death (GO:0043067)	<0.01
APOPTOSIS	extrinsic apoptotic signaling pathway (GO:0097191)	<0.01
APOPTOSIS	regulation of apoptotic process (GO:0042981)	<0.01
APOPTOSIS	intrinsic apoptotic signaling pathway in response to DNA damage (GO:0008630)	<0.01
APOPTOSIS	intrinsic apoptotic signaling pathway (GO:0097193)	<0.01
APOPTOSIS	Fas signaling pathway (GO:0036337)	0.03
APOPTOSIS	canonical Wnt signaling pathway involved in negative regulation of apoptotic process (G	0.04
**AUTOPHAGY**	autophagy (GO:0006914)	<0.01
AUTOPHAGY	process utilizing autophagic mechanism (GO:0061919)	<0.01
**CELL ADHESION**	positive regulation of cell-cell adhesion (GO:0022409)	<0.01
CELL ADHESION	negative regulation of cell junction assembly (GO:1901889)	<0.01
CELL ADHESION	regulation of adherens junction organization (GO:1903391)	0.01
**CELL PROLIFERATION**	cell cycle (GO:0007049)	<0.01
CELL PROLIFERATION	negative regulation of cell population proliferation (GO:0008285)	<0.01
CELL PROLIFERATION	negative regulation of epithelial cell proliferation (GO:0050680)	0.02
**CIRCADIAN RHYTHM ASSOCIATED MECHANISMS**	circadian regulation of gene expression (GO:0032922)	<0.01
CIRCADIAN RHYTHM	entrainment of circadian clock (GO:0009649)	<0.01
CIRCADIAN RHYTHM	photoperiodism (GO:0009648)	0.01
**EXOCYTOSIS**	regulated exocytosis (GO:0045055)	<0.01
EXOCYTOSIS	exocytosis (GO:0006887)	<0.01
EXOCYTOSIS	vesicle-mediated transport (GO:0016192)	<0.01
**IMMUNE RESPONSE**	immune system process (GO:0002376)	<0.01
IMMUNE RESPONSE	response to lipopolysaccharide (GO:0032496)	<0.01
IMMUNE RESPONSE	response to molecule of bacterial origin (GO:0002237)	<0.01
IMMUNE RESPONSE	immune response (GO:0006955)	<0.01
IMMUNE RESPONSE	regulation of immune system process (GO:0002682)	<0.01
IMMUNE RESPONSE	leukocyte activation (GO:0045321)	<0.01
IMMUNE RESPONSE	cellular response to biotic stimulus (GO:0071216)	<0.01
IMMUNE RESPONSE	cellular response to lipopolysaccharide (GO:0071222)	<0.01
IMMUNE RESPONSE	cellular response to molecule of bacterial origin (GO:0071219)	<0.01
IMMUNE RESPONSE	myeloid leukocyte activation (GO:0002274)	<0.01

IMMUNE RESPONSE	positive regulation of immune system process (GO:0002684)	<0.01
IMMUNE RESPONSE	myeloid cell activation involved in immune response (GO:0002275)	<0.01
IMMUNE RESPONSE	myeloid leukocyte mediated immunity (GO:0002444)	<0.01
IMMUNE RESPONSE	neutrophil mediated immunity (GO:0002446)	<0.01
IMMUNE RESPONSE	hematopoietic or lymphoid organ development (GO:0048534)	<0.01
IMMUNE RESPONSE	myeloid cell differentiation (GO:0030099)	<0.01
IMMUNE RESPONSE	granulocyte activation (GO:0036230)	<0.01
IMMUNE RESPONSE	negative regulation of immune system process (GO:0002683)	<0.01
IMMUNE RESPONSE	leukocyte degranulation (GO:0043299)	<0.01
IMMUNE RESPONSE	neutrophil degranulation (GO:0043312)	<0.01
IMMUNE RESPONSE	immune system development (GO:0002520)	<0.01
IMMUNE RESPONSE	leukocyte activation involved in immune response (GO:0002366)	<0.01
IMMUNE RESPONSE	cell activation involved in immune response (GO:0002263)	<0.01
IMMUNE RESPONSE	regulation of macrophage activation (GO:0043030)	<0.01
IMMUNE RESPONSE	myeloid leukocyte differentiation (GO:0002573)	<0.01
IMMUNE RESPONSE	immune effector process (GO:0002252)	<0.01
IMMUNE RESPONSE	neutrophil activation involved in immune response (GO:0002283)	<0.01
IMMUNE RESPONSE	defense response (GO:0006952)	<0.01
IMMUNE RESPONSE	response to other organism (GO:0051707)	<0.01
IMMUNE RESPONSE	leukocyte differentiation (GO:0002521)	<0.01
IMMUNE RESPONSE	positive regulation of T cell activation (GO:0050870)	<0.01
IMMUNE RESPONSE	macrophage activation (GO:0042116)	<0.01
IMMUNE RESPONSE	regulation of T cell activation (GO:0050863)	<0.01
IMMUNE RESPONSE	cellular response to leukemia inhibitory factor (GO:1990830)	<0.01
IMMUNE RESPONSE	response to leukemia inhibitory factor (GO:1990823)	<0.01
IMMUNE RESPONSE	cellular response to interleukin-4 (GO:0071353)	<0.01
IMMUNE RESPONSE	positive regulation of macrophage activation (GO:0043032)	<0.01
IMMUNE RESPONSE	positive regulation of regulatory T cell differentiation (GO:0045591)	0.01
IMMUNE RESPONSE	lymphocyte activation (GO:0046649)	0.01
IMMUNE RESPONSE	B cell activation (GO:0042113)	0.01
IMMUNE RESPONSE	regulation of interleukin-17 production (GO:0032660)	0.01
IMMUNE RESPONSE	alpha-beta T cell activation (GO:0046631)	0.01
IMMUNE RESPONSE	positive regulation of humoral immune response (GO:0002922)	0.01
IMMUNE RESPONSE	CD40 signaling pathway (GO:0023035)	0.02

IMMUNE RESPONSE	regulation of T-helper 1 type immune response (GO:0002825)	0.02
IMMUNE RESPONSE	regulation of dendritic cell differentiation (GO:2001198)	0.02
IMMUNE RESPONSE	regulation of innate immune response (GO:0045088)	0.02
IMMUNE RESPONSE	regulation of interleukin-12 production (GO:0032655)	0.02
IMMUNE RESPONSE	regulation of antigen processing and presentation (GO:0002577)	0.03
IMMUNE RESPONSE	positive regulation of T cell cytokine production (GO:0002726)	0.03
IMMUNE RESPONSE	complement component C5a signaling pathway (GO:0038178)	0.03
IMMUNE RESPONSE	memory T cell activation (GO:0035709)	0.03
IMMUNE RESPONSE	negative regulation of natural killer cell mediated cytotoxicity directed against tumor ce	0.03
IMMUNE RESPONSE	negative regulation of natural killer cell mediated immune response to tumor cell (GO:0	0.03
IMMUNE RESPONSE	negative regulation of immune response to tumor cell (GO:0002838)	0.03
IMMUNE RESPONSE	negative regulation of response to tumor cell (GO:0002835)	0.03
IMMUNE RESPONSE	negative regulation of interleukin-13 production (GO:0032696)	0.03
IMMUNE RESPONSE	B cell differentiation (GO:0030183)	0.03
IMMUNE RESPONSE	regulation of regulatory T cell differentiation (GO:0045589)	0.03
IMMUNE RESPONSE	defense response to virus (GO:0051607)	0.03
IMMUNE RESPONSE	germinal center formation (GO:0002467)	0.04
IMMUNE RESPONSE	B cell apoptotic process (GO:0001783)	0.04
IMMUNE RESPONSE	adaptive immune response (GO:0002250)	0.04
IMMUNE RESPONSE	regulation of immunoglobulin production (GO:0002637)	0.04
IMMUNE RESPONSE	B cell proliferation (GO:0042100)	0.04
IMMUNE RESPONSE	T cell apoptotic process (GO:0070231)	0.04
IMMUNE RESPONSE	monocyte activation (GO:0042117)	0.04
IMMUNE RESPONSE	positive regulation of interleukin-17 production (GO:0032740)	0.05
IMMUNE RESPONSE	regulation of T-helper 17 cell differentiation (GO:2000319)	0.05
IMMUNE RESPONSE	type 2 immune response (GO:0042092)	0.05
IMMUNE RESPONSE	leukocyte migration (GO:0050900)	<0.01
IMMUNE RESPONSE	somatic diversification of immune receptors (GO:0002200)	0.03
**INFLAMMATORY RESPONSE**	leukocyte chemotaxis (GO:0030595)	<0.01
INFLAMMATORY RESPONSE	regulation of cytokine production (GO:0001817)	<0.01
INFLAMMATORY RESPONSE	response to cytokine (GO:0034097)	<0.01
INFLAMMATORY RESPONSE	inflammatory response (GO:0006954)	<0.01
INFLAMMATORY RESPONSE	cellular response to cytokine stimulus (GO:0071345)	<0.01
INFLAMMATORY RESPONSE	positive regulation of cytokine production (GO:0001819)	<0.01

INFLAMMATORY RESPONSE	cytokine-mediated signaling pathway (GO:0019221)	<0.01
INFLAMMATORY RESPONSE	myeloid leukocyte migration (GO:0097529)	<0.01
INFLAMMATORY RESPONSE	regulation of MAPK cascade (GO:0043408)	<0.01
INFLAMMATORY RESPONSE	negative regulation of cytokine secretion (GO:0050710)	<0.01
INFLAMMATORY RESPONSE	granulocyte migration (GO:0097530)	<0.01
INFLAMMATORY RESPONSE	regulation of macrophage migration (GO:1905521)	<0.01
INFLAMMATORY RESPONSE	response to tumor necrosis factor (GO:0034612)	<0.01
INFLAMMATORY RESPONSE	negative regulation of interleukin-6 secretion (GO:1900165)	<0.01
INFLAMMATORY RESPONSE	regulation of inflammatory response (GO:0050727)	<0.01
INFLAMMATORY RESPONSE	regulation of interleukin-6 production (GO:0032675)	<0.01
INFLAMMATORY RESPONSE	cellular response to interleukin-1 (GO:0071347)	<0.01
INFLAMMATORY RESPONSE	chemokine-mediated signaling pathway (GO:0070098)	0.01
INFLAMMATORY RESPONSE	response to interleukin-1 (GO:0070555)	0.01
INFLAMMATORY RESPONSE	regulation of tumor necrosis factor secretion (GO:1904467)	0.01
INFLAMMATORY RESPONSE	regulation of tumor necrosis factor biosynthetic process (GO:0042534)	0.01
INFLAMMATORY RESPONSE	regulation of p38MAPK cascade (GO:1900744)	0.01
INFLAMMATORY RESPONSE	regulation of interleukin-8 production (GO:0032677)	0.02
INFLAMMATORY RESPONSE	positive regulation of interferon-gamma production (GO:0032729)	0.02
INFLAMMATORY RESPONSE	wound healing involved in inflammatory response (GO:0002246)	0.02
INFLAMMATORY RESPONSE	regulation of interferon-gamma-mediated signaling pathway (GO:0060334)	0.02
INFLAMMATORY RESPONSE	regulation of response to interferon-gamma (GO:0060330)	0.02
INFLAMMATORY RESPONSE	regulation of prostaglandin secretion (GO:0032306)	0.02
INFLAMMATORY RESPONSE	regulation of transforming growth factor beta production (GO:0071634)	0.02
INFLAMMATORY RESPONSE	positive regulation of tumor necrosis factor (ligand) superfamily member 11 production	0.03
INFLAMMATORY RESPONSE	positive regulation of interleukin-10 production (GO:0032733)	0.03
INFLAMMATORY RESPONSE	inflammatory response to wounding (GO:0090594)	0.03
INFLAMMATORY RESPONSE	regulation of transforming growth factor beta receptor signaling pathway (GO:0017015	0.03
INFLAMMATORY RESPONSE	regulation of macrophage colony-stimulating factor signaling pathway (GO:1902226)	0.04
**METABOLISM**	regulation of metabolic process (GO:0019222)	<0.01
METABOLISM	regulation of cellular metabolic process (GO:0031323)	<0.01
METABOLISM	regulation of primary metabolic process (GO:0080090)	<0.01
METABOLISM	regulation of macromolecule metabolic process (GO:0060255)	<0.01
METABOLISM	metabolic process (GO:0008152)	<0.01
METABOLISM	macromolecule metabolic process (GO:0043170)	<0.01

METABOLISM	positive regulation of nitrogen compound metabolic process (GO:0051173)	<0.01
METABOLISM	nitrogen compound metabolic process (GO:0006807)	<0.01
METABOLISM	regulation of protein metabolic process (GO:0051246)	<0.01
METABOLISM	regulation of steroid biosynthetic process (GO:0050810)	<0.01
METABOLISM	positive regulation of hormone biosynthetic process (GO:0046886)	<0.01
METABOLISM	regulation of vitamin metabolic process (GO:0030656)	<0.01
METABOLISM	regulation of vitamin D biosynthetic process (GO:0060556)	<0.01
METABOLISM	positive regulation of proteasomal ubiquitin-dependent protein catabolic process (GO:[illegible]	<0.01
METABOLISM	positive regulation of hormone metabolic process (GO:0032352)	<0.01
METABOLISM	positive regulation of vitamin D biosynthetic process (GO:0060557)	0.01
METABOLISM	regulation of cholesterol metabolic process (GO:0090181)	0.01
METABOLISM	mannose metabolic process (GO:0006013)	0.02
METABOLISM	positive regulation of fatty acid transport (GO:2000193)	0.02
METABOLISM	negative regulation of ATP metabolic process (GO:1903579)	0.02
METABOLISM	IMP metabolic process (GO:0046040)	0.03
METABOLISM	positive regulation of lipase activity (GO:0060193)	0.03
METABOLISM	positive regulation of RNA metabolic process (GO:0051254)	<0.01
METABOLISM	DNA metabolic process (GO:0006259)	<0.01
METABOLISM	RNA metabolic process (GO:0016070)	<0.01
METABOLISM	cellular detoxification of aldehyde (GO:0110095)	0.03
METABOLISM	androgen metabolic process (GO:0008209)	0.02
**NERVOUS SYSTEM**	microglial cell migration (GO:1904124)	0.03
NERVOUS SYSTEM	detection of chemical stimulus involved in sensory perception of smell (GO:0050911)	<0.01
NERVOUS SYSTEM	detection of stimulus involved in sensory perception (GO:0050906)	<0.01
NERVOUS SYSTEM	detection of chemical stimulus involved in sensory perception (GO:0050907)	<0.01
NERVOUS SYSTEM	detection of stimulus (GO:0051606)	<0.01
NERVOUS SYSTEM	sensory perception of smell (GO:0007608)	<0.01
NERVOUS SYSTEM	sensory perception of chemical stimulus (GO:0007606)	<0.01
NERVOUS SYSTEM	nervous system process (GO:0050877)	<0.01
NERVOUS SYSTEM	sensory perception (GO:0007600)	<0.01
NERVOUS SYSTEM	regulation of gliogenesis (GO:0014013)	<0.01
NERVOUS SYSTEM	regulation of neuroinflammatory response (GO:0150077)	<0.01
NERVOUS SYSTEM	regulation of microglial cell migration (GO:1904139)	<0.01
NERVOUS SYSTEM	trans-synaptic signaling (GO:0099537)	<0.01

NERVOUS SYSTEM	chemical synaptic transmission (GO:0007268)	<0.01
NERVOUS SYSTEM	anterograde trans-synaptic signaling (GO:0098916)	<0.01
NERVOUS SYSTEM	positive regulation of microglial cell migration (GO:1904141)	<0.01
NERVOUS SYSTEM	positive regulation of glial cell migration (GO:1903977)	<0.01
NERVOUS SYSTEM	synaptic signaling (GO:0099536)	<0.01
NERVOUS SYSTEM	regulation of amyloid-beta clearance (GO:1900221)	<0.01
NERVOUS SYSTEM	regulation of neuron death (GO:1901214)	0.01
NERVOUS SYSTEM	regulation of neurogenesis (GO:0050767)	0.01
NERVOUS SYSTEM	regulation of oligodendrocyte differentiation (GO:0048713)	0.01
NERVOUS SYSTEM	regulation of neuron apoptotic process (GO:0043523)	0.02
NERVOUS SYSTEM	axonogenesis (GO:0007409)	0.03
NERVOUS SYSTEM	regulation of amyloid-beta formation (GO:1902003)	0.03
NERVOUS SYSTEM	axon guidance (GO:0007411)	0.03
NERVOUS SYSTEM	neuron projection guidance (GO:0097485)	0.03
NERVOUS SYSTEM	glial cell activation (GO:0061900)	0.04
NERVOUS SYSTEM	positive regulation of synaptic transmission (GO:0050806)	0.05
**TISSUE REMODELING and MORPHOGENESIS**	regulation of tissue remodeling (GO:0034103)	<0.01
TISSUE REMODELING and MORPHOGENESIS	positive regulation of tissue remodeling (GO:0034105)	<0.01
TISSUE REMODELING and MORPHOGENESIS	regulation of bone remodeling (GO:0046850)	<0.01
TISSUE REMODELING and MORPHOGENESIS	regulation of bone resorption (GO:0045124)	<0.01
TISSUE REMODELING and MORPHOGENESIS	osteoclast differentiation (GO:0030316)	<0.01
TISSUE REMODELING and MORPHOGENESIS	regulation of epithelial cell migration (GO:0010632)	<0.01
TISSUE REMODELING and MORPHOGENESIS	regulation of ossification (GO:0030278)	<0.01
TISSUE REMODELING and MORPHOGENESIS	regulation of osteoblast differentiation (GO:0045667)	0.01
TISSUE REMODELING and MORPHOGENESIS	intestinal epithelial cell development (GO:0060576)	0.02
TISSUE REMODELING and MORPHOGENESIS	columnar/cuboidal epithelial cell maturation (GO:0002069)	0.03
TISSUE REMODELING and MORPHOGENESIS	regulation of epithelial to mesenchymal transition (GO:0010717)	0.04
TISSUE REMODELING and MORPHOGENESIS	mammary gland duct morphogenesis (GO:0060603)	0.04
TISSUE REMODELING and MORPHOGENESIS	branching involved in mammary gland duct morphogenesis (GO:0060444)	0.01
TISSUE REMODELING and MORPHOGENESIS	connective tissue replacement (GO:0097709)	0.01
TISSUE REMODELING and MORPHOGENESIS	regulation of muscle hypertrophy (GO:0014743)	0.05
TISSUE REMODELING and MORPHOGENESIS	wound healing (GO:0042060)	<0.01
TISSUE REMODELING and MORPHOGENESIS	response to wounding (GO:0009611)	<0.01
**TRANSCRIPTION**	regulation of nucleic acid-templated transcription (GO:1903506)	<0.01

TRANSCRIPTION	mitochondrial transcription (GO:0006390)	0.03
**VASCULAR SYSTEM**	regulation of endothelial cell differentiation (GO:0045601)	<0.01
VASCULAR SYSTEM	positive regulation of vascular endothelial growth factor receptor signaling pathway (G(	<0.01
VASCULAR SYSTEM	regulation of angiogenesis (GO:0045765)	0.01
VASCULAR SYSTEM	regulation of establishment of endothelial barrier (GO:1903140)	0.01
VASCULAR SYSTEM	regulation of vascular smooth muscle cell proliferation (GO:1904705)	0.02
VASCULAR SYSTEM	negative regulation of blood vessel morphogenesis (GO:2000181)	0.02
VASCULAR SYSTEM	response to angiotensin (GO:1990776)	0.02
VASCULAR SYSTEM	blood vessel maturation (GO:0001955)	0.04
VASCULAR SYSTEM	response to ischemia (GO:0002931)	0.04
VASCULAR SYSTEM	blood coagulation (GO:0007596)	<0.01
VASCULAR SYSTEM	coagulation (GO:0050817)	<0.01
VASCULAR SYSTEM	hemostasis (GO:0007599)	<0.01
VASCULAR SYSTEM	platelet degranulation (GO:0002576)	<0.01
**RESPONSE TO STIMULUS**	cellular response to stimulus (GO:0051716)	<0.01
RESPONSE TO STIMULUS	response to oxygen-containing compound (GO:1901700)	<0.01
RESPONSE TO STIMULUS	response to starvation (GO:0042594)	<0.01
RESPONSE TO STIMULUS	cellular response to nutrient levels (GO:0031669)	<0.01
RESPONSE TO STIMULUS	cellular response to oxidative stress (GO:0034599)	<0.01
RESPONSE TO STIMULUS	cellular response to amino acid starvation (GO:0034198)	<0.01
RESPONSE TO STIMULUS	response to hormone (GO:0009725)	0.01
RESPONSE TO STIMULUS	response to steroid hormone (GO:0048545)	0.02
RESPONSE TO STIMULUS	response to UV (GO:0009411)	0.02
RESPONSE TO STIMULUS	cellular response to manganese ion (GO:0071287)	0.02
RESPONSE TO STIMULUS	cellular response to hydrogen peroxide (GO:0070301)	0.03
RESPONSE TO STIMULUS	cellular response to progesterone stimulus (GO:0071393)	0.04
RESPONSE TO STIMULUS	response to clozapine (GO:0097338)	0.04
RESPONSE TO STIMULUS	response to lipid (GO:0033993)	<0.01
RESPONSE TO STIMULUS	response to stress (GO:0006950)	<0.01
RESPONSE TO STIMULUS	cellular response to stress (GO:0033554)	<0.01
RESPONSE TO STIMULUS	regulation of response to stress (GO:0080134)	<0.01
**REPRODUCTIVE SYSTEM/REPRODUCTION**	embryonic placenta development (GO:0001892)	<0.01
REPRODUCTIVE SYSTEM/REPRODUCTION	fertilization (GO:0009566)	0.03
REPRODUCTIVE SYSTEM/REPRODUCTION	tube development (GO:0035295)	0.03

REPRODUCTIVE SYSTEM/REPRODUCTION	single fertilization (GO:0007338)	0.02
**OTHERS**	regulation of kinase activity (GO:0043549)	<0.01
	regulation of protein serine/threonine kinase activity (GO:0071900)	<0.01
	regulation of developmental process (GO:0050793)	<0.01
	regulation of body fluid levels (GO:0050878)	<0.01
	regulation of protein secretion (GO:0050708)	<0.01
	growth hormone secretion (GO:0030252)	0.02
	DNA damage checkpoint (GO:0000077)	0.02
	negative regulation of telomere maintenance via telomere lengthening (GO:1904357)	0.03
	positive regulation of fever generation (GO:0031622)	0.03
	iron ion transport (GO:0006826)	0.03
	regulation of phosphorylation (GO:0042325)	<0.01
	protein modification process (GO:0036211)	<0.01
