# Supplementary material for: Gene Expression Profiling in Fibromyalgia Indicates an Autoimmune Origin of the Disease and Opens New Avenues for Targeted Therapy
Source: J Clin Med. 2020 Jun 10;9(6):1814. doi: 10.3390/jcm9061814 (PMC7356177; doi:10.3390/jcm9061814)
Supplement: Supplementary file 1 [file jcm-09-01814-s001.zip › Supplementary table 3.pdf]

	Biological pathway	*p-value*
**Apoptosis**	TRAIL signaling pathway	<0.01
	p53 pathway	0.01
	Direct p53 effectors	0.03
**Circadian rhythm**	Circadian rhythm pathway	<0.01
	Circadian Clock	0.02
**Endocrine system**	Plasma membrane estrogen receptor signaling	<0.01
**Immune response**	IL3-mediated signaling events	<0.01
	S1P1 pathway	<0.01
	IL5-mediated signaling events	<0.01
	CDC42 signaling events	<0.01
	Regulation of CDC42 activity	<0.01
	IL12-mediated signaling events	<0.01
	Cytokine Signaling in Immune system	<0.01
	Signaling by Interleukins	<0.01
	IL23-mediated signaling events	0.01
	LPA receptor mediated events	0.01
	CD40/CD40L signaling	0.02
	TCR signaling in naive CD4+ T cells	0.02
	Immune System	0.02
	IL2 signaling events mediated by PI3K	0.04
**Inflammatory response**	GMCSF-mediated signaling events	<0.01
	AP-1 transcription factor network	<0.01
	PIP3 activates AKT signaling	<0.01
	TGFBR	<0.01
	Interleukin-1 signaling	<0.01
	Alternative NF-kappaB pathway	0.01
	Interleukin-1 processing	0.01
	Canonical NF-kappaB pathway	0.01
	CXCR4-mediated signaling events	0.02
	TNF receptor signaling pathway	0.02
	Toll Like Receptor 10 (TLR10) Cascade	0.02
	Toll Like Receptor 7/8 (TLR7/8) Cascade	0.03
	p38 MAPK signaling pathway	0.03

	Toll Like Receptor 9 (TLR9) Cascade	0.03
	MyD88 cascade initiated on plasma membrane	0.04
	TNF alpha/NF-kB	0.04
	Toll Like Receptor 5 (TLR5) Cascade	0.05
**Metabolism**	LKB1 signaling events	<0.01
	Arf6 signaling events	<0.01
	mTOR signaling pathway	<0.01
	Insulin Pathway	<0.01
	IGF1 pathway	<0.01
	cholesterol biosynthesis I	0.03
	cholesterol biosynthesis III (via desmosterol)	0.03
	cholesterol biosynthesis II (via 24,25-dihydrolanosterol)	0.03
	Beta-oxidation of pristanoyl-CoA	0.03
**Neuronal system**	Signalling by NGF	<0.01
	p75(NTR)-mediated signaling	0.01
	NGF signalling via TRKA from the plasma membrane	0.02
**Pain perception and transmis**	IFN-gamma pathway	<0.01
	Class I PI3K signaling events	<0.01
	Class I PI3K signaling events mediated by Akt	<0.01
	G-protein beta:gamma signalling	0.01
	Enkephalin release	0.03
**Vascular system**	ErbB receptor signaling network	<0.01
	VEGF and VEGFR signaling network	<0.01
	PAR1-mediated thrombin signaling events	<0.01
	Thrombin/protease-activated receptor (PAR) pathway	<0.01
	PDGF receptor signaling network	<0.01
	Internalization of ErbB1	<0.01
	Urokinase-type plasminogen activator (uPA) and uPAR-mediated signaling	<0.01
	EGFR-dependent Endothelin signaling events	<0.01
	Endothelins	<0.01
	GAB1 signalosome	<0.01
	ALK1 signaling events	<0.01
	PAR4-mediated thrombin signaling events	0.01
	Thromboxane signalling through TP receptor	0.03

Integrins in angiogenesis	0.03
Signaling by EGFR	0.04
