# Supplementary material for: Gene Expression Profiling in Fibromyalgia Indicates an Autoimmune Origin of the Disease and Opens New Avenues for Targeted Therapy
Source: J Clin Med. 2020 Jun 10;9(6):1814. doi: 10.3390/jcm9061814 (PMC7356177; doi:10.3390/jcm9061814)
Supplement: Supplementary file 1 [file jcm-09-01814-s001.zip › Supplementary table 4.pdf]

Genes included in the six modules

M1	M2	M3	M4	M5	M6
ASB6	C5AR1	ADAM8	ALYREF	ADAM17	ARL8A
CDC16	CCL20	AKAP9	AREG	AGFG1	ATM
CUL1	CCL3	ATP6V0C	B7RP1	AP2A2	BBS10
DZIP3	CCL4L1	BORA	BCAS2	ASH2L	BHLHE40
FBXL4	CD40LG	BUB3	BCL2L11	BIVM-ERCC5	BRI3
FBXO11	CNR2	CBX4	BET1	BNIP1	CASP9
KBTBD8	CSF1	CCL7	BUD31	BTLA	CCNT1
KLHL21	CX3CR1	CD93	CBX5	C12orf65	CEBPB
LMO7	CXCL16	CEACAM1	CDKN1A	CCNE1	CHEK1
LONRF1	CXCL3	CENPC	CDKN1B	CCNH	CRY1
MYLIP	CXCR6	CENPJ	CLP1	CD27	CRY2
RNF111	FPR1	CEP162	COMMD10	CD58	CTDP1
RNF114	GNB2	CEP192	COMMD5	CDC45	CTPS1
RNF138	GNG11	CEP290	COMMD6	CEBPA	CTPS2
RNF19A	GNG2	CEP70	CPSF2	CREB1	DDIT3
RNF34	GNG5	CEP97	CSTF1	CTSB	EAF1
SIAH1	GPR18	CFD	CTNNB1	DNM3	EIF5
SMURF1	GPR37L1	CLEC12A	CWC22	EXOSC10	FAM120B
SOCS1	IFNG	CLEC5A	CWC27	FAM20C	FEN1
TRIM39	IL1B	CLU	DBF4	FAS	IPO8
UBA3	IL1R1	CSNK1D	DPY30	FLT1	JUND
UBE2A	ITGAX	DNMT3A	ETF1	FOSB	KCNQ5
UBE2H	P2RY12	EZH2	FEM1C	FURIN	LPCAT1
UBE2M	PF4	F13A1	FOXO3	GNA12	MED27
UBE2S	PPBP	FERMT3	GATAD2A	GNA13	MLLT3
UBE2V2	S1PR1	HDAC1	GP1BA	GNA15	MRPL57
	TAS2R14	HDAC5	GRIA1	GNAZ	MRPS23
	TLR10	HIST1H2AB	HIF1A	GNL1	MRPS5
	TLR7	HIST1H2AE	HSPA5	H2AFX	NFIL3
	TNFRSF1B	HIST1H2BB	ITGA2B	HAVCR2	PDF
	TNFSF11	HIST1H2BC	LEF1	HBEGF	PER1
		HIST1H2BD	NFKB2	JAG1	PER2
		HIST1H2BF	ORC3	KIF1B	PLEC
		HIST1H2BG	ORC5	KIF20B	PRMT2
		HIST1H2BI	ORC6	LDLRAP1	PRMT3
		HIST1H2BL	PPARG	LTBP1	RPL7A
		HIST1H2BO	PPIE	MAFB	RPS14
		HIST1H3A	PPIL1	MAPKAPK2	RPS26
		HIST1H4F	PPP2R2A	MCM3	RPS3
		HIST2H2AA	PRC1	MCM6	SRP19
		HIST2H2AA3	RAB1A	MEIS1	THRAP3
		HIST2H2BE	SEC23IP	MRPL12	TTC37
		HIST2H3D	SEC24A	MRPL17	UTP6
		IL10	SF3B3	MSH2	

IL16	SMG1	MTIF2
IL1A	SPP1	MTIF3
IL6	SRC	NAT10
ITGB3BP	SRRT	NBAS
KDM6B	TCF7	P4HB
KIF2A	TNFRSF4	PLCB1
LILRB2	TNFSF4	PNO1
MGAM	TRAF5	POLR3B
MMP9	TRAPPC4	PSMA4
MMRN1	TRAPPC6B	RPA2
NBEAL2	UPF1	SDC2
NDEL1	USO1	SEC61A2
NFKB1	ZEB1	SNX9
NHLRC2		STON2
NOD2		SURF4
NSL1		TFRC
NUP43		TIMP1
ORM2		TNFRSF12A
PHC2		TRMT1L
PLK4		TRMT6
PPP2CA		UBTF
PRKAR2B		WASL
RANBP2		WDR3
RANGAP1		XPC
RCC2		
RELB		
RPGRIP1L		
SEC13		
SELP		
SIGLEC14		
SKA2		
SLC11A1		
SPARC		
SVIP		
THBS1		
TNF		
TRAF3		
TUBA4A		
TUBB4B		
TUBGCP4		
VEGFA		
