# Supplementary material for: Gene Expression Profiling in Fibromyalgia Indicates an Autoimmune Origin of the Disease and Opens New Avenues for Targeted Therapy
Source: J Clin Med. 2020 Jun 10;9(6):1814. doi: 10.3390/jcm9061814 (PMC7356177; doi:10.3390/jcm9061814)
Supplement: Supplementary file 1 [file jcm-09-01814-s001.zip › Supplementary table 5.pdf]

Fold Change	Gene Symbol	miRNA target	Description
22.6	RP3-496C20.1	no miRNA target	novel transcript, antisense to FAM83F
19.68	RP11-449G16.1	no miRNA target	novel transcript, sense intronic to MAP4K3
13.86	RP11-166B2.3	no miRNA target	novel transcript sense intronic to GSPT1
12.34	AC017002.2	no miRNA target	novel transcript
9.45	RP11-769O8.3	no miRNA target	novel variant, sense intronic toYES1
8.19	RP11-656E20.5	no miRNA target	novel transcript
7.76	ASAP1-IT1	no miRNA target	ASAP1 intronic transcript 1
7.16	RP11-242C19.2	no miRNA target	novel transcript, sense intronic to GK
6.47	RP1-168P16.2	no miRNA target	novel transcript
6.18	LINC01272	no miRNA target	long intergenic non-protein coding RNA 1272
5.88	RP11-134L10.1	no miRNA target	novel transcript antisense to C7orf49
5.69	RP11-138A9.1	no miRNA target	novel transcript
5.63	LOC100133331	no miRNA target	uncharacterized LOC100133331
5.38	RP11-274J7.2	no miRNA target	novel transcript
5.37	CATIP-AS1	no miRNA target	CATIP antisense RNA 1
5.25	RC3H1-IT1	no miRNA target	RC3H1 intronic transcript 1 (non-protein coding)
5.23	CTD-2651B20.6	2 miRNAs targets	novel transcript
5.2	CTB-131B5.2	no miRNA target	novel transcript
4.82	LINC-PINT	no miRNA target	long intergenic non-protein coding RNA, p53 induced transcript
4.76	RP11-327F22.6	no miRNA target	novel transcript
4.65	RP1-151F17.1	15 miRNAs targets	novel transcript
4.56	RP11-380G5.2	no miRNA target	Jeck2013 ALT_ACCEPTOR, ALT_DONOR, coding, INTERNAL,
4.31	RP11-318K15.2	no miRNA target	putative novel transcript
4.25	RP11-707G18.1	no miRNA target	novel transcript
4.22	RP11-44N11.1	no miRNA target	novel transcript
4.21	CTC-428H11.2	no miRNA target	novel transcript antisense to RASA1
4.18	RP11-333J10.2	no miRNA target	novel transcript, sense intronic AATF
4.14	LINC01001	no miRNA target	long intergenic non-protein coding RNA 1001
4.1	GK-AS1	no miRNA target	GK antisense RNA 1 [Source:HGNC Symbol
4.09	RP11-151N17.1	no miRNA target	novel transcript
-4.31	RP4-607I7.1	no miRNA target	novel transcript
-4.32	RP11-61O1.2	no miRNA target	novel transcript
-4.35	AC093609.1	no miRNA target	novel gene

-4.43	WDR11-AS1	no miRNA target	WDR11 antisense RNA 1
-4.52	AC009299.3	29 miRNAs targets	novel transcript
-4.54	LINC00861	no miRNA target	long intergenic non-protein coding RNA 861
-4.74	RP11-730K11.1	no miRNA target	novel transcript, antisense to SORL1
-5.05	RP11-283I3.6	1 mirna target	novel transcript
-5.18	PRKCQ-AS1	no miRNA target	PRKCQ antisense RNA 1
-5.32	RP4-791M13.3	no miRNA target	novel transcript
-5.88	AC093642.4	no miRNA target	novel transcript
-6.71	CHRM3-AS2	no miRNA target	CHRM3 antisense RNA 2
-6.8	RP11-18H21.1	no miRNA target	novel transcript
-6.86	LINC00402	no miRNA target	long intergenic non-protein coding RNA 402
-8.47	RP11-190C22.9	no miRNA target	novel transcript, antisense to ADPRH
-9	RP11-747H7.3	8 miRNAs targets	novel transcript, intronic to CATSPERB
-9.1	RP5-1180E21.5	no miRNA target	novel transcript, antisense to CEPT1
-9.37	LINC01550	no miRNA target	long intergenic non-protein coding RNA 1550
-9.48	LINC00989	no miRNA target	long intergenic non-protein coding RNA 989
-9.53	RP11-664D1.1	no miRNA target	novel transcript
-10.68	RP11-160E2.11	no miRNA target	novel transcript
-10.69	KCNJ2-AS1	no miRNA target	KCNJ2 antisense RNA 1 (head to head)
-11.07	AC084082.3	no miRNA target	novel transcript

uc063agg.1
