# Supplementary material for: Gene Expression Profiling in Fibromyalgia Indicates an Autoimmune Origin of the Disease and Opens New Avenues for Targeted Therapy
Source: J Clin Med. 2020 Jun 10;9(6):1814. doi: 10.3390/jcm9061814 (PMC7356177; doi:10.3390/jcm9061814)
Supplement: Supplementary file 1 [file jcm-09-01814-s001.zip › Supplementary table 6.pdf]

miRNAs targets of the selected LncRNAs				
**CTD-2651B20.6**	**RP1-151F17.1**	**AC009299.3**	**RP11-283I3.6**	**RP11-747H7.3**
hsa-miR-375	hsa-mir-186-3p	hsa-miR-143-3p	hsa-miR-1306-3p	hsa-mir-130a-3p
hsa-miR-214-5p	hsa-mir-148a	hsa-miR-376a-3p		hsa-miR-23a-3p
	hsa-mir-148b	hsa-miR-376b-3p		hsa-mir-23b-3p
	hsa-miR-152	hsa-miR-425-5p		hsa-miR-23c
	hsa-miR-214-5p	hsa-miR-4770		hsa-miR-323a-3p
	hsa-miR-219-5p	hsa-miR-6088		hsa-miR-6835-3p
	hsa-miR-4782-5p	hsa-let-7a-2		hsa-miR-135a2
	hsa-miR-493-5p	hsa-let-7b-5p		hsa-mir-367-3p
	hsa-mir-6766	hsa-let-7c		
	hsa-miR-758-3p	hsa-let-7d-3p		
	hsa-miR-7-2-3p	hsa-let-7e-3p		
	hsa-miR-200b-5p	hsa-let-7f-5p		
	hsa-mir-200c-3p	hsa-let-7g-5p		
	hsa-miR-3129-5p	hsa-let-7i-5p		
	hsa-miR-429	hsa-miR-1271-3p		
		hsa-miR-182-3p		
		hsa-miR-376c-3p		
		hsa-miR-4458		
		hsa-miR-4500		
		hsa-miR-495-3p		
		hsa-miR-5688		
		hsa-miR-6835-3p		
		hsa-miR-96-5p		
		hsa-mir-98		
		hsa-mir-142-3p		
		hsa-miR-340-5p		
		hsa-miR-378c		
		hsa-miR-422a		
		hsa-miR-5590-3p		

Genes modulated in FM that are targeted by miRNA targets of the five selected LncRNA				
**CTD-2651B20.6**	**RP1-151F17.1**	**AC009299.3**	**RP11-283I3.6**	**RP11-747H7.3**
BUB3	AGFG1	ABCA1	none	ALAS1

C14orf1	ANKRD17	ABHD17C		ARHGEF6
CRY2	ASF1A	AEN		ASAH2B
HMG20A	BAG6	AHCYL1		ASF1A
JAG1	BUB3	ANKIB1		BET1
KDM2A	C14orf1	ANKRD46		BORA
KLF5	CABLES1	ARHGAP6		C18orf54
NHEJ1	CCNJ	ARHGEF6		C1orf52
NXF1	CDKN1B	ARRDC4		CBX1
PGS1	CNEP1R1	ASB6		CCL7
UBXN2B	CNN3	BCL2L11		CCNH
WASL	CRY2	BCL2L2		CRLF3
	CSRP2	BIN3		CSTF1
	DDIT4	BLOC1S5		DTWD1
	ERRFI1	BSDC1		DUSP5
	FAM8A1	C2orf88		EFHD2
	GART	C5orf51		FAM168B
	GNA12	CAB39L		FAXDC2
	GNG11	CABLES1		GAD1
	GPBP1L1	CAMKK2		GOLPH3L
	GRAP2	CASP2		HERPUD1
	HEMGN	CBX1		IRF2
	HMG20A	CCL3		KLF10
	HMGB3	CCL3L3		LMO7
	HSPA13	CCL7		MEIS1
	IER5	CCNJ		MICU3
	ING2	CCNJL		MINPP1
	ITPK1	CD200R1		N4BP1
	JAG1	CDKN1A		NEK7
	JKAMP	CEBPB		NEU1
	KDM2A	CEP97		NFYB
	KIAA0040	CLP1		NLK
	KLF10	CNEP1R1		NRGN
	KLF4	CPD		PATL1
	KLF5	CPEB2		PI4K2A

MMD	CRLF3	PKIA
MSL2	CRY2	PLEKHF2
MXD4	CSRNP1	PPIF
NHEJ1	CSTF1	RPS3
NLK	CTPS1	SEC23IP
NOG	CTSB	SPRY2
NUDCD1	CUL4B	SPTSSB
NXF1	CWC22	SUGT1
PAPSS2	DDIT3	SYS1
PCMTD1	DENND2C	TFRC
PGRMC1	DTWD1	TMED5
PGS1	DUSP16	TMEM2
PKIA	DUSP4	TMEM68
PPP2CA	DYNC1I2	TNFSF11
PPP2R2A	EEA1	TOP1
PRKACB	EFHD2	TRIB1
PRKAR2B	EPB41L3	UBE2H
PSIP1	ERCC4	WHAMM
RAB21	EXOSC10	YES1
RGS10	FAS	ZC3H13
RHOQ	FNIP2	ZNF225
RNF214	FOSL2	ZNF292
RPAP3	GCNT4	
SDC2	GGA2	
SH2B3	GINM1	
SLC25A16	GNA13	
SMARCAD1	GNG5	
SURF4	GPCPD1	
SYS1	GPR132	
TFAM	GPR180	
TFRC	GRIA1	
TMEM106B	GRPEL2	
TNFAIP8	HBEGF	
TSC22D1	HERPUD1	

UBXN2B	HES1
VEGFA	HS2ST1
WASL	HSPA5
WHAMM	IL10
XKR8	INSIG2
ZEB1	IRF9
	IRG1
	ITGA6
	ITGB3
	JMJD6
	KIF2A
	KLF10
	KLF9
	LDLRAD4
	LIMK2
	LMLN
	MAP2K3
	MAP3K1
	MAPK6
	MAPK7
	MBLAC2
	MBNL1
	MBTPS2
	MED1
	MEF2D
	MFAP3L
	MGA
	MINPP1
	MXD1
	NEK7
	NFE2L2
	NFKBIZ
	NHLRC2
	NKX3-1

PAPSS2
PATL1
PCGF5
PDCD10
PDE12
PET117
PGRMC1
PHF6
PHLDA2
PIGA
PIGU
PKIA
PPIL1
PPP2R2A
PRKACB
PTP4A1
RBM41
RCC2
RGS16
RHEB
RHOQ
RIOK3
RIT1
RPS3
S1PR1
SBDS
SCAMP1
SCYL3
SDAD1
SDC2
SENP2
SH2B3
SKIL
SLC25A16

SLC25A32
SLC30A1
SLC30A6
SLC35A1
SMAD7
SMARCAD1
SOCS1
SPIN1
SPTSSB
SSX2IP
SUGT1
SUPT20H
SURF4
TAF10
TET1
THBS1
TMEM2
TMEM68
TMX1
TNFRSF1B
TRAF3
TRANK1
TRIB1
TSC22D3
UAP1
UBE2H
UBXN2B
UGCG
USP42
USP44
USP45
VPS37B
WASL
XKR8

YTHDF3
ZBTB10
ZBTB26
ZBTB5
ZC4H2
ZEB1
ZNF10
ZNF146
ZNF148
ZNF391
ZNF583
ZNF585A
