# Supplementary material for: Gene Expression Profiling in Fibromyalgia Indicates an Autoimmune Origin of the Disease and Opens New Avenues for Targeted Therapy
Source: J Clin Med. 2020 Jun 10;9(6):1814. doi: 10.3390/jcm9061814 (PMC7356177; doi:10.3390/jcm9061814)
Supplement: Supplementary file 1 [file jcm-09-01814-s001.zip › Supplementary table 7.pdf]

Module associated targeted FM genes

Targeted FM genes	Module	LncRNA
BUB3	M3	CTD2651B20.6
CRY2	M6	CTD2651B20.6
JAG1	M5	CTD2651B20.6
WASL	M5	CTD2651B20.6
AGFG1	M5	RP1-151F17.1
BUB3	M3	RP1-151F17.1
CDKN1B	M4	RP1-151F17.1
CRY2	M6	RP1-151F17.1
GNA12	M5	RP1-151F17.1
GNG11	M2	RP1-151F17.1
JAG1	M5	RP1-151F17.1
PPP2CA	M3	RP1-151F17.1
PPP2R2A	M4	RP1-151F17.1
PRKAR2B	M3	RP1-151F17.1
SDC2	M5	RP1-151F17.1
SURF4	M5	RP1-151F17.1
TFRC	M5	RP1-151F17.1
VEGFA	M3	RP1-151F17.1
WASL	M5	RP1-151F17.1
ZEB1	M4	RP1-151F17.1
ADB6	M1	AC009299.3
BCL2L11	M4	AC009299.3
CCL3	M2	AC009299.3
CCL7	M3	AC009299.3
CDKN1A	M4	AC009299.3
CEBPB	M6	AC009299.3
CEP97	M3	AC009299.3
CLP1	M4	AC009299.3
CRY2	M6	AC009299.3
CSTF1	M4	AC009299.3
CTPS1	M6	AC009299.3
CTSB	M5	AC009299.3

CWC22	M4	AC009299.3
DDIT3	M6	AC009299.3
EXOSC10	M5	AC009299.3
FAS	M5	AC009299.3
GNA13	M5	AC009299.3
GNG5	M2	AC009299.3
GRIA1	M4	AC009299.3
HBEGF	M5	AC009299.3
HSPA5	M4	AC009299.3
IL10	M3	AC009299.3
KIF2A	M3	AC009299.3
NHLRC2	M3	AC009299.3
PPIL1	M4	AC009299.3
PPP2R2A	M4	AC009299.3
RCC2	M3	AC009299.3
RPS3	M6	AC009299.3
S1PR1	M2	AC009299.3
SDC2	M5	AC009299.3
SOCS1	M1	AC009299.3
SURF4	M5	AC009299.3
THBS1	M3	AC009299.3
TNFRSF1B	M2	AC009299.3
TRAF3	M3	AC009299.3
UBE2H	M1	AC009299.3
WASL	M5	AC009299.3
ZEB1	M4	AC009299.3
BET1	M4	RP11-747H7.3
BORA	M3	RP11-747H7.3
CCL7	M3	RP11-747H7.3
CCNH	M5	RP11-747H7.3
CSTF1	M4	RP11-747H7.3
LMO7	M1	RP11-747H7.3
MEIS1	M5	RP11-747H7.3
RPS3	M6	RP11-747H7.3

SEC23IP	M4	RP11-747H7.3
TFRC	M5	RP11-747H7.3
TNFSF11	M2	RP11-747H7.3
UBE2H	M1	RP11-747H7.3
