# Supplementary material for: Gene Expression Profiling in Fibromyalgia Indicates an Autoimmune Origin of the Disease and Opens New Avenues for Targeted Therapy
Source: J Clin Med. 2020 Jun 10;9(6):1814. doi: 10.3390/jcm9061814 (PMC7356177; doi:10.3390/jcm9061814)
Supplement: Supplementary file 1 [file jcm-09-01814-s001.zip › Supplementary table 8.pdf]

RHEUMATOID ARTHRITIS

Biological Pathway	*p-value*
Arf6 signalling events	0.040
Class I PI3K signalling events	0.040
Class I PI3K signalling events mediated by Akt	0.040
EGFR-dependent Endothelin signalling events	0.041
Endothelins	0.040
ErbB receptor signalling network	0.045
GMCSF-mediated signalling events	0.025
IFN-gamma pathway	0.028
IGF1 pathway	0.044
IL3-mediated signalling events	0.027
IL5-mediated signalling events	0.045
Insulin Pathway	0.040
Internalization of ErbB1	0.040
mTOR signalling pathway	0.040
PAR1-mediated thrombin signalling events	0.031
PDGF receptor signalling network	0.047
Plasma membrane oestrogen receptor signalling	0.033
S1P1 pathway	0.040
Thrombin/protease-activated receptor (PAR) pathway	0.032
Urokinase-type plasminogen activator (uPA) and uPAR-mediat	0.040
VEGF and VEGFR signalling network	0.055
Alpha9 beta1 integrin signalling events	0.020
Arf6 downstream pathway	0.040
Arf6 trafficking events	0.040
Beta1 integrin cell surface interactions	0.026
EGF receptor (ErbB1) signalling pathway	0.040
ErbB1 downstream signalling	0.040
Glypican 1 network	0.056
Glypican pathway	0.055
Integrin family cell surface interactions	0.032
Integrin-linked kinase signalling	0.053
Nectin adhesion pathway	0.049
PDGFR-beta signalling pathway	0.040
Proteoglycan syndecan-mediated signalling events	0.006
Signalling events mediated by focal adhesion kinase	0.040
Signalling events mediated by hepatocyte growth factor recep	0.047
Signalling events mediated by VEGFR1 and VEGFR2	0.051
Sphingosine 1-phosphate (S1P) pathway	0.045
Syndecan-1-mediated signalling events	0.032

FYBROMIALGIA

Biological pathway	*p-value*
Arf6 signaling events	<0.01

Class I PI3K signaling events	<0.01
Class I PI3K signaling events mediated by Akt	<0.01
EGFR-dependent Endothelin signaling events	<0.01
Endothelins	<0.01
ErbB receptor signaling network	<0.01
GMCSF-mediated signaling events	<0.01
IFN-gamma pathway	<0.01
IGF1 pathway	<0.01
IL3-mediated signaling events	<0.01
IL5-mediated signaling events	<0.01
Insulin Pathway	<0.01
Internalization of ErbB1	<0.01
mTOR signaling pathway	<0.01
PAR1-mediated thrombin signaling events	<0.01
PDGF receptor signaling network	<0.01
Plasma membrane estrogen receptor signaling	<0.01
S1P1 pathway	<0.01
Thrombin/protease-activated receptor (PAR) pathway	<0.01
Urokinase-type plasminogen activator (uPA) and uPAR-mediat	<0.01
VEGF and VEGFR signaling network	<0.01
ALK1 signaling events	<0.01
Alternative NF-kappaB pathway	0.01
AP-1 transcription factor network	<0.01
Beta-oxidation of pristanoyl-CoA	0.03
Canonical NF-kappaB pathway	0.01
CD40/CD40L signaling	0.02
CDC42 signaling events	<0.01
cholesterol biosynthesis I	0.03
cholesterol biosynthesis II (via 24,25-dihydrolanosterol)	0.03
cholesterol biosynthesis III (via desmosterol)	0.03
Circadian Clock	0.02
Circadian rhythm pathway	<0.01
CXCR4-mediated signaling events	0.01
Cytokine Signaling in Immune system	<0.01
Direct p53 effectors	0.03
Enkephalin release	0.03
GAB1 signalosome	<0.01
G-protein beta:gamma signalling	0,01
IL12-mediated signaling events	<0.01
IL2 signaling events mediated by PI3K	0.04
IL23-mediated signaling events	0.01
Immune System	0.02
Integrins in angiogenesis	0.03
Interleukin-1 processing	0.01
Interleukin-1 signaling	<0.01
LKB1 signaling events	<0.01
LPA receptor mediated events	0.01

MyD88 cascade initiated on plasma membrane	0.04
NGF signalling via TRKA from the plasma membrane	0.02
p38 MAPK signaling pathway	0.03
p53 pathway	0.01
p75(NTR)-mediated signaling	0.01
PAR4-mediated thrombin signaling events	0.01
PIP3 activates AKT signaling	<0.01
Regulation of CDC42 activity	<0.01
Signaling by EGFR	0.04
Signaling by Interleukins	<0.01
Signalling by NGF	<0.01
TCR signaling in naive CD4+ T cells	0.02
TGFBR	<0.01
Thromboxane signalling through TP receptor	0.03
TNF alpha/NF-kB	0.04
TNF receptor signaling pathway	0.02
Toll Like Receptor 10 (TLR10) Cascade	0.02
Toll Like Receptor 5 (TLR5) Cascade	0.05
Toll Like Receptor 7/8 (TLR7/8) Cascade	0.03
Toll Like Receptor 9 (TLR9) Cascade	0.03
TRAIL signaling pathway	<0.01
