# Supplementary figures and images for: Gene Expression Profiling in Fibromyalgia Indicates an Autoimmune Origin of the Disease and Opens New Avenues for Targeted Therapy
Source: J Clin Med. 2020 Jun 10;9(6):1814. doi: 10.3390/jcm9061814 (PMC7356177; doi:10.3390/jcm9061814)

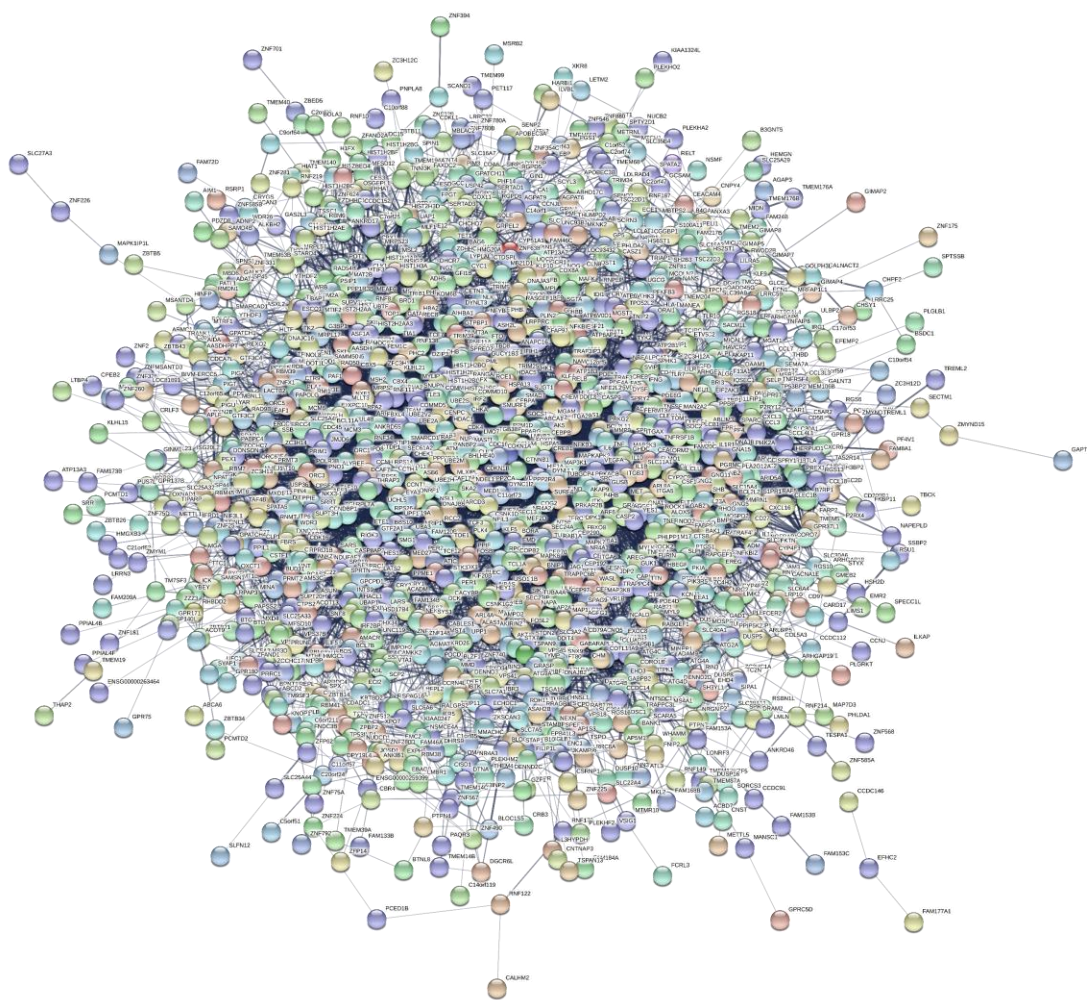

Supplement: Supplementary file 1 [file jcm-09-01814-s001.zip › Supplementary figure 1.pdf]

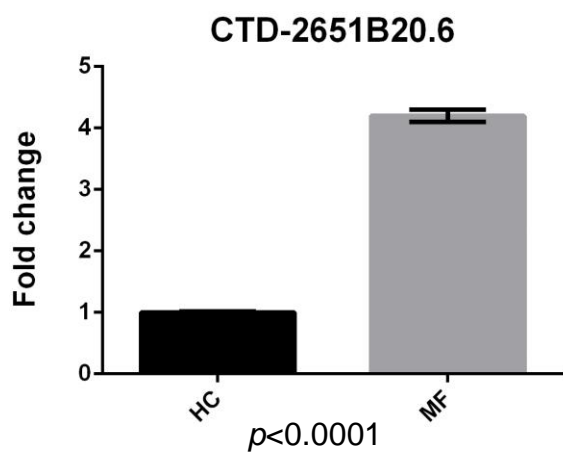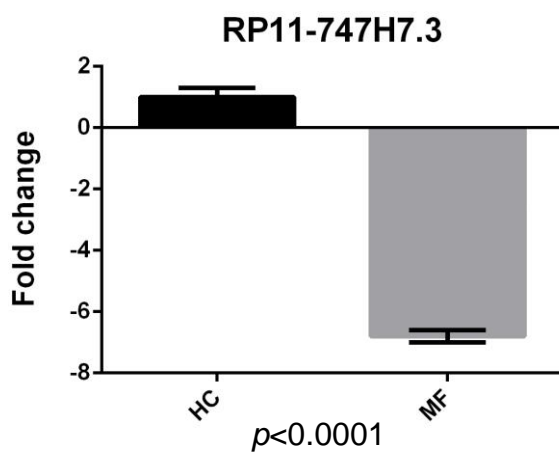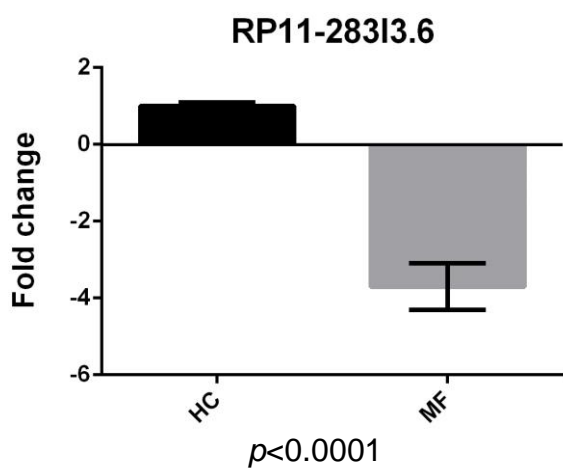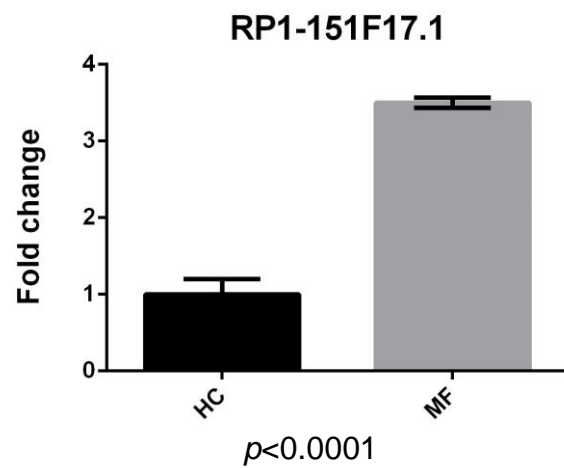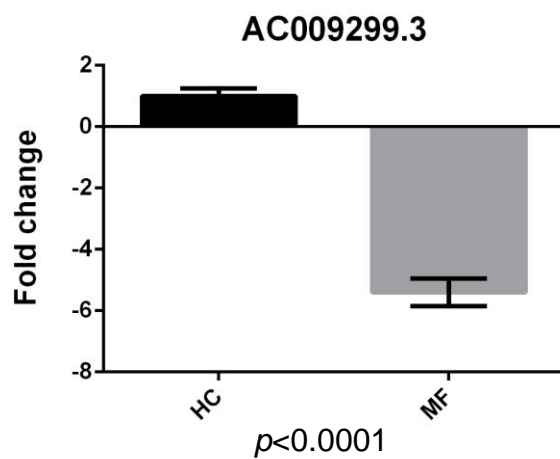

Supplement: Supplementary file 1 [file jcm-09-01814-s001.zip › Supplementary figure 2 def.pdf]

**TGF-beta**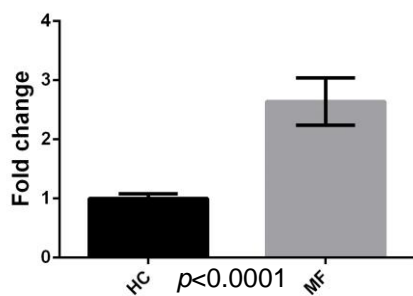**IL-6**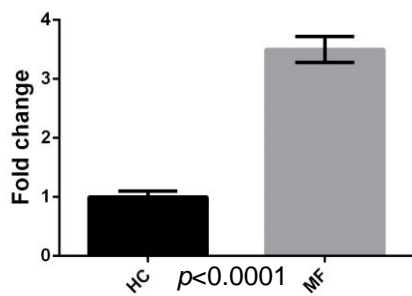**IL-21**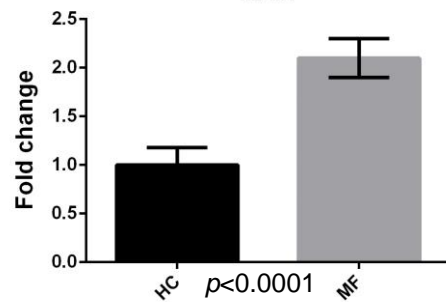**IL-23**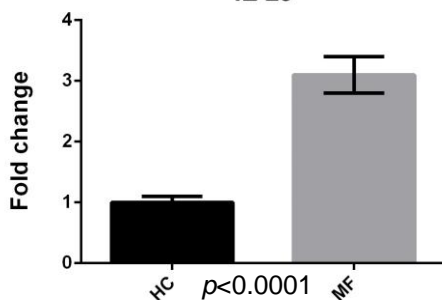**IL-17**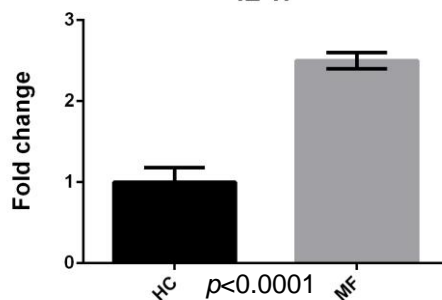**TNF**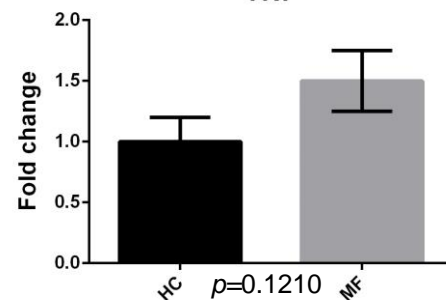**IL-10**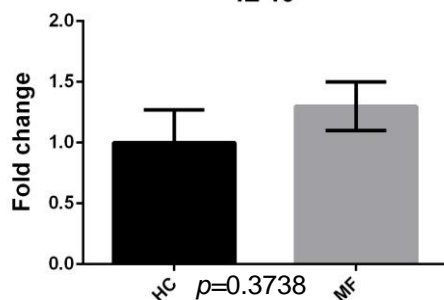**IL-8**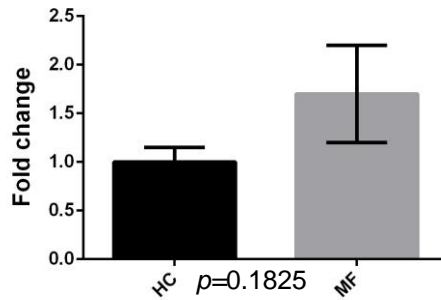**IL-1**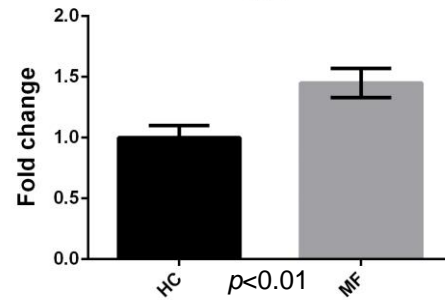**IL-2**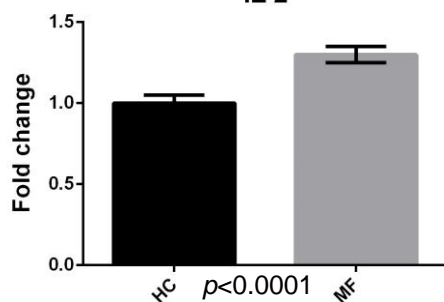**IL-4**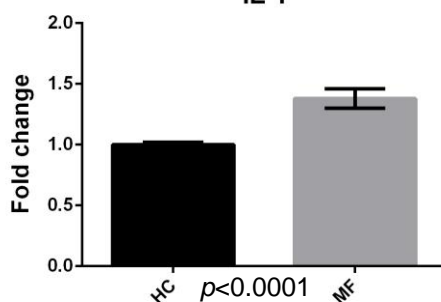**Tie-2**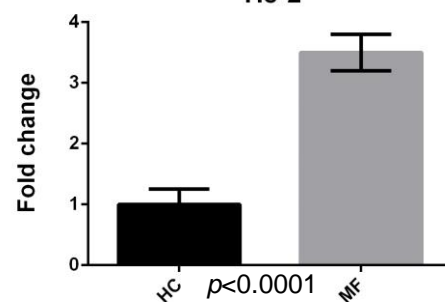**TARC**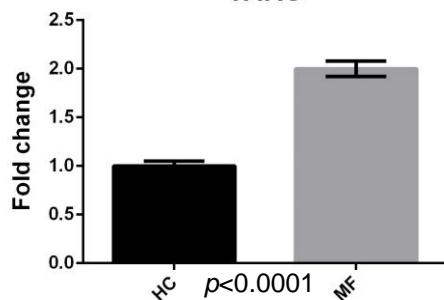**SAA**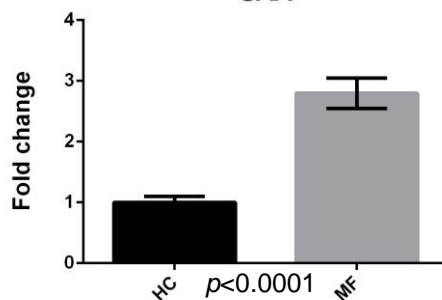

Supplement: Supplementary file 1 [file jcm-09-01814-s001.zip › Supplementary figure 3 def.pdf]
